# PG1058 Is a Novel Multidomain Protein Component of the Bacterial Type IX Secretion System

**DOI:** 10.1371/journal.pone.0164313

**Published:** 2016-10-06

**Authors:** Jacqueline E. Heath, Christine A. Seers, Paul D. Veith, Catherine A. Butler, Nor A. Nor Muhammad, Yu-Yen Chen, Nada Slakeski, Benjamin Peng, Lianyi Zhang, Stuart G. Dashper, Keith J. Cross, Steven M. Cleal, Caroline Moore, Eric C. Reynolds

**Affiliations:** Oral Health Cooperative Research Centre, Melbourne Dental School, Bio21 Institute, The University of Melbourne, Melbourne, Australia; East Carolina University Brody School of Medicine, UNITED STATES

## Abstract

*Porphyromonas gingivalis* utilises the Bacteroidetes-specific type IX secretion system (T9SS) to export proteins across the outer membrane (OM), including virulence factors such as the gingipains. The secreted proteins have a conserved carboxy-terminal domain essential for type IX secretion that is cleaved upon export. In *P*. *gingivalis* the T9SS substrates undergo glycosylation with anionic lipopolysaccharide (A-LPS) and are attached to the OM. In this study, comparative analyses of 24 Bacteroidetes genomes identified ten putative novel components of the T9SS in *P*. *gingivalis*, one of which was PG1058. Computer modelling of the PG1058 structure predicted a novel N- to C-terminal architecture comprising a tetratricopeptide repeat (TPR) domain, a β-propeller domain, a carboxypeptidase regulatory domain-like fold (CRD) and an OmpA_C-like putative peptidoglycan binding domain. Inactivation of *pg1058* in *P*. *gingivalis* resulted in loss of both colonial pigmentation and surface-associated proteolytic activity; a phenotype common to T9SS mutants. Immunoblot and LC-MS/MS analyses of subcellular fractions revealed T9SS substrates accumulated within the *pg1058* mutant periplasm whilst whole-cell ELISA showed the Kgp gingipain was absent from the cell surface, confirming perturbed T9SS function. Immunoblot, TEM and whole-cell ELISA analyses indicated A-LPS was produced and present on the *pg1058* mutant cell surface although it was not linked to T9SS substrate proteins. This indicated that PG1058 is crucial for export of T9SS substrates but not for the translocation of A-LPS. PG1058 is a predicted lipoprotein and was localised to the periplasmic side of the OM using whole-cell ELISA, immunoblot and LC-MS/MS analyses of subcellular fractions. The structural prediction and localisation of PG1058 suggests that it may have a role as an essential scaffold linking the periplasmic and OM components of the T9SS.

## Introduction

Chronic periodontitis is an inflammatory disease characterised by accretion of a polymicrobial biofilm (subgingival plaque) on the tooth, destruction of the supporting tissues of the teeth and ultimately tooth loss [1]. Periodontitis has been linked with systemic diseases including cardiovascular disease, rheumatoid arthritis, diabetes, preterm birth and low birth weight [2, 3]. The presence of *Porphyromonas gingivalis*, *Tannerella forsythia*, and *Treponema denticola* in the subgingival plaque is strongly associated with the clinical indicators of disease [4, 5]. In particular, subgingival plaque composed of greater than 10–15% *P*. *gingivalis* cells is a predictor of imminent disease progression [6]. In addition, this Gram-negative, black-pigmented, anaerobic bacterium has been described as a keystone pathogen of the disease, which through dysregulation of the local immune response disrupts homeostasis causing dysbiosis and disease progression [7–9].

*P*. *gingivalis* produces a variety of surface-associated virulence factors implicated in pathogenesis, including lipopolysaccharide (LPS), fimbriae, capsular polysaccharide, haemagglutinin (HagA) and the gingipains [10]. The gingipains are Lys-specific (Kgp) and Arg-specific (RgpA and RgpB) cysteine proteinases, considered to be the bacterium’s major virulence factors responsible for ~85% of its proteolytic activity, and are hence the most studied [8, 11–14].

The gingipains belong to a group of 34 surface-associated proteins in *P*. *gingivalis* with a conserved carboxy-terminal domain (CTD) [15]. The CTD has been suggested to contain a C-terminal secretion signal as when removed from the proteins RgpB and haemin binding protein HBP35 they were not secreted from the periplasm [15–17]. This was corroborated by the secretion and modification of green fluorescent protein when it was fused with the CTD sequence from either RgpB, CPG70, peptidylarginine deiminase (PAD) or P27 [17, 18].

The CTD-family proteins are also known to be extensively glycosylated [19–21]. This modification is recognised by the monoclonal antibody MAb 1B5 [19]. MAb 1B5 recognises a phosphorylated branched mannan epitope (Manα1–2 Manα1-phosphate) found in anionic polysaccharide (APS), the polysaccharide repeating unit of the anionic lipopolysaccharide (A-LPS) produced by *P*. *gingivalis* [22]. The A-LPS modification is covalently linked to the mature C-terminus of the CTD-family proteins and has been proposed to be responsible for tethering them to the cell surface [23, 24].

Several *P*. *gingivalis* proteins have been shown to have a role in the secretion and modification of CTD-family proteins. Mutations in the genes coding PorT [18], Sov [25] and LptO (PorV/PG27) [26] resulted in a non-pigmented phenotype and reduced or no gingipain activity, indicative of defective secretion. CTD-family proteins and homologues of *porT* are present in some members of the Bacteroidetes phylum such as *Cytophaga hutchinsonii*, but absent from *Bacteroides thetaiotaomicron* [27]. Comparative genome analysis of genes present in *P*. *gingivalis* and *C*. *hutchinsonii* but absent in *B*. *thetaiotaomicron* was conducted to identify those coding for proteins with possible involvement in CTD-family protein secretion. Fifty-five genes were identified of which gene inactivation and phenotype analyses revealed *porK*, *porL*, *porM*, *porN*, *porP*, *porQ*, *porU (pg0026)*, *porW*, *porX* and *porY* to code components of the secretion system, with accumulation of CTD-family proteins in the periplasm and decreased gingipain activity associated with cells and culture supernatants of the mutants [27]. The PorSS (Por secretion system) identified by Sato *et al*. [27], now commonly referred to as the type IX secretion system (T9SS), is responsible for the OM secretion and surface-association of CTD-family proteins in Bacteroidetes spp., utilising the Sec pathway for inner membrane (IM) transit [17, 27–29].

Although several proteins have been identified as components of the T9SS, knowledge of their actual function is limited. PorX and PorY have been suggested to be a putative response regulator and a putative histidine kinase respectively, of a two-component signal transduction system and were shown to regulate *porT*, *sov*, *porK*, *porL*, *porM*, *porN* and *porP* expression in *P*. *gingivalis* [27, 30]. Recent work has shown a direct interaction between recombinant PorX and PorY proteins, with rPorY exhibiting histidine kinase activity [31]. Furthermore, PorK and PorN have recently been shown to interact to form large ring-shaped complexes of 50 nm diameter. These rings appeared to be located on the periplasmic face of the OM and were suggested to be part of the translocation apparatus [32].

*P*. *gingivalis* W50 and ATCC 33277 mutants lacking LptO accumulate A-LPS and unmodified substrates in the periplasm [33]. Mass spectrometry showed *P*. *gingivalis* W50 possesses tetra- and penta-acylated forms of mono-phosphorylated lipid A whilst the *lptO* mutant exhibited only penta-acylated mono-phosphorylated lipid A consistent with its accumulation in the periplasm prior to surface deacylation [33]. A *P*. *gingivalis* ATCC 33277 *porT* mutant showed a similar phenotype to the *lptO* mutant in regards to the accumulation of substrates in the periplasm, except that the *porT* mutant exhibited a lipid A profile similar to that of wild-type [33]. This indicated that LptO is required for the normal O-deacylation of mono-phosphorylated lipid A in *P*. *gingivalis*, suggesting that LptO is essential for the normal translocation of A-LPS and the coordinated secretion/attachment of T9SS substrates [33].

PorU (PG0026), a surface-associated CTD-family protein which lacks glycosylation, was determined to be a signal peptidase responsible for the cleavage of the CTD from the other T9SS substrates in *P*. *gingivalis* [34]. Although T9SS substrates largely accumulated in the periplasm of a *pg0026 (porU)* mutant, of those that were surface-exposed the CTD was not cleaved from the precursor proteins and the proteins were not modified with A-LPS [34].

Recently analysis of a *wbaP* mutant that is defective in A-LPS synthesis showed that T9SS substrates were secreted into the culture fluid with their CTDs cleaved. In place of the CTD sequences were a variety of amino acids and peptides from the growth medium [23]. This suggested that CTD-cleavage and modification of the T9SS substrates by PorU occurs through a sortase-like mechanism [23]. It was also revealed that LptO associates with PorU, suggesting that the integral OMP LptO is involved in tethering PorU to the cell surface [35, 36]. Furthermore, the close association of these two proteins could provide the link between A-LPS translocation and presentation to the CTD sortase to anchor the T9SS substrate proteins at the cell surface.

Although several proteins have been established as essential components of the T9SS with some localised within the cell, the precise functions of most of the components remain unknown. To explore whether all components of the T9SS in *P*. *gingivalis* have been identified we extended the bioinformatic approach of Sato *et al*. [27] and report the identification of several novel putative T9SS components and demonstrate experimentally that one of these is important for the function of the T9SS in *P*. *gingivalis*.

## Results

### Bioinformatic prediction of T9SS components

Identification of T9SS components was previously conducted by Sato *et al*. [27], using a genome comparison approach of *P*. *gingivalis* and *C*. *hutchinsonii*, which possess CTD-family proteins, and *B*. *thetaiotaomicron* which does not [27]. To identify additional T9SS components this bioinformatic approach was extended whereby the genomes of twenty-four Bacteroidetes spp. were examined using BLASTp, for the ability to code for proteins which are also coded by the *P*. *gingivalis* W83 genome. The species were separated into those coding for CTD-family proteins (CTD-positive) and those which did not (CTD-negative). Thirty-seven *P*. *gingivalis* W83 proteins with an average BLASTp score at least two times greater in the CTD-positive species than in the CTD-negative species were shortlisted. The list was further refined to include twenty-nine candidates based on BLAST verification of the species distribution (**Table 1**).

**Table 1:** T9SS components predicted using the differential genomics approach.

Locus Tag^a^		Protein ID^b^	Description	MPS/MNS^c^	Implicated in T9SS	Bioinformatic prediction
W83	ATCC 33277					
PG0026	PGN_0022	PorU	C-terminal signal peptidase	10.6	[27, 34]	[27]
PG0027	PGN_0023	PorV/LptO	T9SS Protein V	7.8	[26, 33]	This study
PG0052	PGN_2001	PorY	Sensor histidine kinase	3.6	[27]	[27]
PG0133	PGN_0246		c protein; putative exopolysaccharaide biosynthesis	3.8		[27]
PG0162	PGN_0274		RNA polymerase subunit sigma-24 factor	2.8	[37]	This study
PG0236	PGN_0341		Right-handed beta helix region domain protein	2.9		[27]
PG0264	PGN_0361		Glycosyl transferase, group 2 family protein	3.1		This study
PG0287	PGN_1677	PorP	T9SS Protein P	3.7	[27]	[27]
PG0288	PGN_1676	PorK	T9SS Protein K	6.2	[27]	[27]
PG0289	PGN_1675	PorL	T9SS Protein L	2.6	[27]	[27]
PG0290	PGN_1674	PorM	T9SS Protein M	4.9	[27]	[27]
PG0291	PGN_1673	PorN	T9SS Protein N	2.8	[27]	This study
PG0441	PGN_1556		TonB-dependent receptor	2.2		This study
PG0534	PGN_1437		TonB-dependent receptor	7.4	[38]	[27]
PG0602	PGN_0645	PorQ	T9SS Protein Q	4.1	[27]	[27]
PG0751	PGN_0778	PorT	T9SS Protein T	3.5	[18]	[27]
PG0809	PGN_0832	Sov	Gliding motility protein	39.6	[25]	[27]
PG0928	PGN_1019	PorX	Chemotaxis protein CheY	8.8	[27]	[27]
PG0945	PGN_1005		ABC transporter permease	8.1		[27]
PG1058	PGN_1296		TPR^d^, WD40^d^, CRD^d^, OmpA family domain	4.2	This study	This study
PG1572	PGN_0538		Membrane protein, putative	2.9		This study
PG1573	PGN_0537		Transcriptional regulator, Crp family	2.3		This study
PG1604	PGN_0509		Immunoreactive 84 kDa antigen PG93[39]	4.3	[21]	[27]
PG1685			PF04338 family protein	3.6		This study
PG1786			PF11276 family protein	2.2		This study
PG1850	PGN_1783		Hypothetical protein	6.3		This study
PG1947	PGN_1877	PorW	T9SS Protein W	7.8	[27]	[27]
PG2071	PGN_2051		Acyltransferase	2.5		This study
PG2092	PGN_0144		Hypothetical protein	4.7		This study

^a^ Locus Tag represented by PG numbers in *P*. *gingivalis* W83 with homologues in *P*. *gingivalis* ATCC 33277 indicated by PGN numbers when appropriate.

^b^ Protein ID indicated where a designation has been made.

^c^ MPS/MNS: Mean CTD-positive species BLAST score / Mean CTD-negative species BLAST score.

^d^ TPR, tetratricopeptide motif; WD40, WD40 motif; CRD, carboxypeptidase regulatory domain-like fold.

This method successfully predicted twelve known Por proteins and Sov, previously considered to be T9SS components. Furthermore, PG0162 and PG0534 predicted by this analysis were previously implicated in secretion whereby mutants exhibited reduced gingipain production when the genes coding for these proteins were inactivated [31, 37, 38]. Prediction of PG1604 herein supports the previous proposition that PG1604 could be a T9SS component due to sequence similarity with the CTD of PorU and the lack of glycosyl modification [21]. PG0133, PG0236 and PG0945 were also previously predicted to be components of the T9SS [27] however no supporting experimental evidence has yet been reported for these proteins. Ten newly identified T9SS candidates remained, one is potentially involved in polysaccharide biosynthesis (PG0264), one in lipid synthesis (PG2071) and one is a putative TonB-linked receptor (PG0441). The remaining seven proteins have no potential function assigned at this stage. PG1058 was the first of these novel proteins selected for further investigation.

### The predicted structure of PG1058

**Fig 1:**
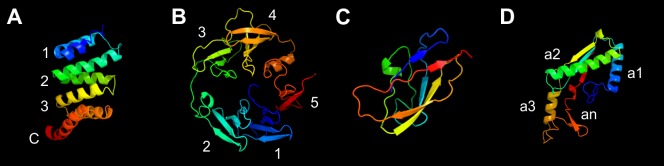
Phyre^2^ modelling of PG1058 predicted structural domains. Structures are coloured from blue at the N-terminus to red at the C-terminus. **A.** A TPR domain with three anti-parallel α-helix repeats (α-helix repeats numbered 1–3) and one capping helix (denoted by C) [47]. PG1058 Lys25-Arg153 modelled at 99.9% confidence against c412wA, the myosin chaperone UNC-45 from *Caenorhabditis elegans* in complex with a Hsp70 peptide [48]. **B.** A five bladed β–propeller domain (blades numbered 1–5). PG1058 Asp167-Ala438 modelled at 99.9% confidence against c2w8bB, *E*. *coli* TolB (a six bladed propeller protein) in complex with Pal [49]. **C.** The CRD. PG1058 Ile443-Arg528 modelled at 99.5% confidence against cmn8A, *Drosophila melanogaster* carboxypeptidase d isoform 1b2 short [50]. **D.** An OmpA_C-like domain. PG1058 Asn536-Val666 modelled at 100.0% confidence against c1r1m1, the OmpA-like domain from RmpM of *N*. *meningitidis* [45] with three α-helices (α-helices numbered α1-α3) and an additional α-helix (αn) which has seven additional residues relative to RmpM which are not represented in this model.

Bioinformatic analyses of the PG1058 sequence using Pfam and the conserved domain database indicated that PG1058 consists of four domains. Beginning from the N-terminus, the first domain contains tetratricopeptide repeats (TPR) (residues 71–131; pfam13432) [40, 41] and the second domain contains a WD40 repeat (residues 226-262, pfam07676) [42, 43]. However prediction with the InterPro database suggests a longer domain encompassing residues 167–433 with five repeats, potentially forming a β–propeller structure. The putative β–propeller domain is followed by a domain with a predicted carboxypeptidase regulatory domain-like fold (CRD; residues 443-525, pfam13620). The C-terminal domain is an OmpA_C-like domain (residues 536-666; CD07185), responsible for the annotation of PG1058 as an OmpA-family protein [44–46]. Thus PG1058 is predicted to be a modular protein. Structure predictions using Phyre^2^ supported the Pfam predictions as indicated by the models generated (**Fig 1, S1 Fig**). PG1058 is predicted to be a lipoprotein due to the presence of an N-terminal type II signal peptide. We know of no characterised protein that has a domain architecture like that predicted for PG1058.

**Fig 2:**
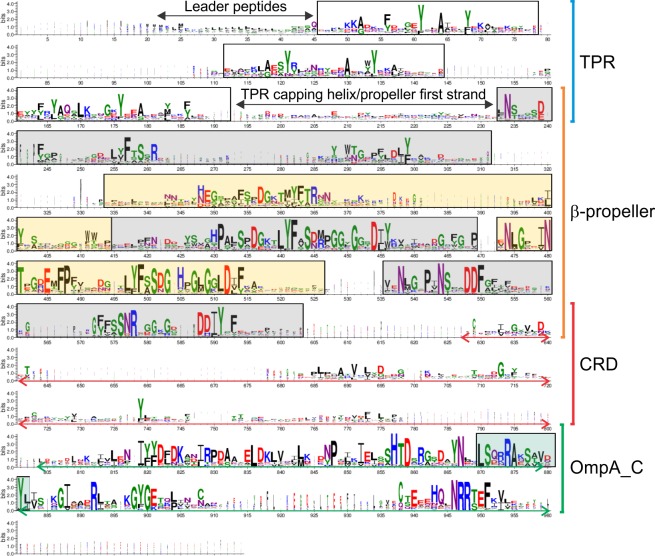
PG1058 sequence conservation. A representative sequence from each of the 70 genera with PG1058 homologues were aligned using CLUSTAL W [51] and a sequence logo was generated using WebLogo 3 [52, 53]. The approximate boundaries of the CRD and OmpA_C-like domains are indicated by arrows. Low homology regions are primarily due to insertions in some sequences, although overall identity in the CRD is lower than seen in the other domains. The three TPR repeats are boxed and the five β–propeller blades are in alternating gray and yellow boxes. The putative peptidoglycan binding motif is boxed in green.

An extensive bioinformatic analysis established that there were 424 proteins within the Bacteroidetes phylum with domain order similarity to PG1058, represented by 174 species from 70 genera across the 4 classes. Interestingly, while many species had one PG1058 homologue, some had multiple PG1058 homologues, ranging from 2 to 13 per species. Furthermore, some homologues had N-terminal and C-terminal extensions relative to PG1058. Multiple sequence alignment using one homologue from each genus (the highest scored BLASTp match to PG1058) when represented as a sequence logo shows highly conserved residues within each domain, especially in the β-propeller and OmpA_C-like domains (**Fig 2**). Alignment of the PG1058 homologues containing regions of additional residues relative to PG1058 showed the same regions of conservation, with the main differences being the N-terminal and C-terminal extensions, a possible domain (or additional blade) between repeat 1 and 2 of the β–propeller, and a longer CRD region due to insertions (*data not shown*).

### Co-transcription of *pg1056*, *pg1057* and *pg1058*

Scrutiny of the *pg1058* locus indicated that it may be the third gene in a three gene operon including *pg1056* and *pg1057*. RT-PCR analysis of *P*. *gingivalis* W50 transcripts confirmed that *pg1058* is indeed part of an operon (**S2A Fig**). A *pg1058* mutant was generated by insertional inactivation with *ermF* in the *P*. *gingivalis* W50 strain. The mutant was complemented *in trans* by chromosomal integration at the *mfa1* locus, of a DNA fragment which contained *pg1058* and *cepA* for recombinant selection. The recombinant was designated *pg1058*^*+*^. Non-endpoint RT-PCR analysis of the W50, *pg1058* and *pg1058*^*+*^ strains indicated that transcription of *pg1056* and *pg1057* was not detrimentally affected in the *pg1058* mutant and *pg1058*^*+*^ complement strains (**S2B Fig**). Thus a polar effect on those genes would be unlikely to account for any mutant phenotype observed.

### The *pg1058* mutant has an altered pigmentation, haemagglutination and proteolytic phenotype

**Fig 3:**
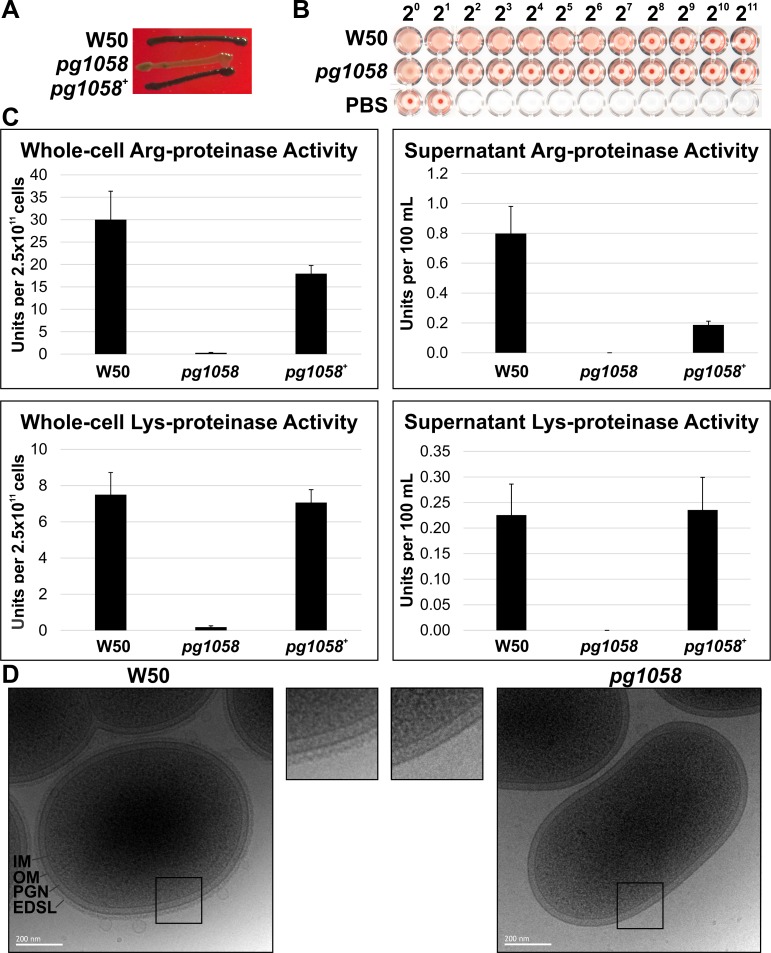
Phenotypic characterisation of the *pg1058* mutant compared to *P*. *gingivalis* W50. **A.** Unlike *P*. *gingivalis* W50, the *pg1058* mutant (*pg1058*) does not produce black pigment when grown on blood agar. The phenotype is restored following *pg1058* complementation (*pg1058*^*+*^). **B.** The *pg1058* mutant showed substantially reduced haemagglutination activity relative to *P*. *gingivalis* W50. Bacteria were 2-fold serially diluted from 2^0^ to 2^11^ in PBS and photographed after incubation with sheep erythrocytes at RT for 3 h. **C.** Arg-specific (indicative of the presence of RgpA and RgpB proteinases) and Lys-specific (indicative of the presence of Kgp proteinase) proteinase activity of W50 (wild-type), *pg1058* mutant and *pg1058*^*+*^ complement strains in whole-cells and culture supernatants. Units per 2.5 x 10^11^ cells equates to amount of substrate hydrolysed in μmol/min/2.5 x 10^11^ cells whilst Units per 100 mL equates to amount of substrate hydrolysed in μmol/min/supernatant derived from 100 mL of culture containing 2.5 x 10^11^ cells. Inactivation of *pg1058* abolished the Arg- and Lys-specific proteolytic activity of the cells, which was restored after *pg1058* complementation. **D.** Absence of EDSL on the *P*. *gingivalis pg1058* mutant. Cryo-EM micrographs representative of the *P*. *gingivalis* W50 and *pg1058* mutant. Outer membrane (OM), inner membrane (IM), peptidoglycan (PGN) and electron dense surface layer (EDSL) are indicated. Scale bar is 200 nm.

*P*. *gingivalis* colonies grown on blood agar appear black due to the accumulation of haemoglobin-derived μ-oxo bishaem on the cell surface, in a process dependent on the proteolytic degradation of haemoglobin by T9SS substrates, the gingipains [54–56]. As such, pigmentation is therefore also dependent on a functioning T9SS to present the gingipains on the cell surface. The *pg1058* mutant exhibited altered colonial pigmentation on blood agar, which was reverted in the complement strain (**Fig 3A**). The ability of *P*. *gingivalis* to haemagglutinate erythrocytes is also linked to appropriate secretion and surface-association of gingipains and the haemagglutinin HagA. The *pg1058* mutant showed a clear reduction in haemagglutination ability relative to W50 (**Fig 3B**). Proteinase activity assays indicated that the *pg1058* mutant had significantly reduced cell-associated Arg- and Lys-specific proteolytic activities (**Fig 3C**). *P*. *gingivalis* W50 has an electron dense surface layer (EDSL) of ~20 nm thickness coating the surface of the cell that is visible in cryo-EM micrographs [33]. This coating has been shown to contain gingipains and other T9SS substrate proteins [23]. The *pg1058* mutant lacked a visible EDSL in cryo-EM (**Fig 3D**) suggesting the absence of gingipains and other T9SS substrates on the cell surface.

### Aberrant localisation of T9SS substrates in the *pg1058* mutant

**Fig 4:**
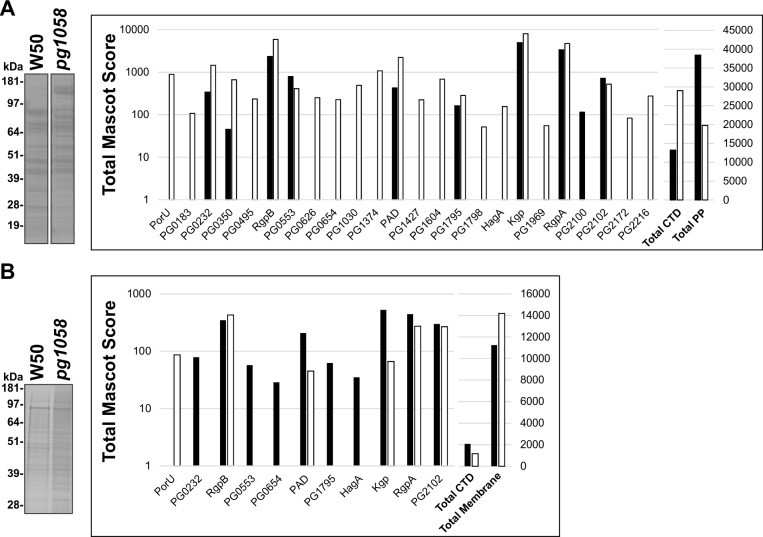
Aberrant localisation of T9SS substrates in the *pg1058* mutant. *P*. *gingivalis* W50 and *pg1058* mutant periplasm (**A**) or total membrane (**B**) fractions were separated via SDS-PAGE and stained with SimplyBlue™ SafeStain. Each lane was divided into segments and analysed by LC-MS/MS. When the same protein was identified from multiple gel segments the Mascot scores were summed. The total Mascot score for the substrates identified from W50 (black bars) and the *pg1058* mutant (white bars) were plotted on a logarithmic axis (left Y-axis). The total Mascot score for the combined substrates and the total Mascot scores for the combined periplasmic or membrane proteins were plotted on a linear axis (right Y-axis). Proteins are indicated by the Locus Tag in the *P*. *gingivalis* W83 strain unless previously designated with a protein ID.

*P*. *gingivalis* W50 and *pg1058* mutant periplasmic fractions were subjected to SDS-PAGE and Coomassie staining which revealed different protein profiles (**Fig 4A**). LC-MS/MS analysis of peptides extracted from the gel identified 255 proteins overall including 24 T9SS substrates and 20 proteins that have been previously localised to the periplasm. An accumulation of T9SS substrates in the periplasm of the *pg1058* mutant was established by comparison of the total Mascot scores of the substrates, with several substrates detected only in the mutant (**Fig 4A**). Using this technique, PorU, both a substrate and component of the T9SS, was not detected in the W50 periplasm however was readily detected in the periplasm of the *pg1058* mutant. This could be explained by accumulation of PorU in the periplasm of the mutant, suggesting that its secretion across the OM was hindered.

Given the identification of T9SS substrates accumulating in the periplasm of the *pg1058* mutant it follows that the OM proteome of the *pg1058* mutant may have altered. Total membrane fractions from W50 and the *pg1058* mutant were therefore also subjected to proteome analysis similar to that performed on the periplasm fractions. In the membranes 278 proteins were identified overall including 11 T9SS substrates. A lower abundance of T9SS substrates in the membrane fraction of the *pg1058* mutant was indicated by a lower total Mascot score for the substrates when compared to W50 (**Fig 4B)**. The accumulation of T9SS substrates in the periplasm with a coincident reduction in the membrane of the *pg1058* mutant in comparison to W50 was consistently observed across multiple biological replicates indicating a perturbation of the T9SS (*data not shown*).

**Fig 5:**
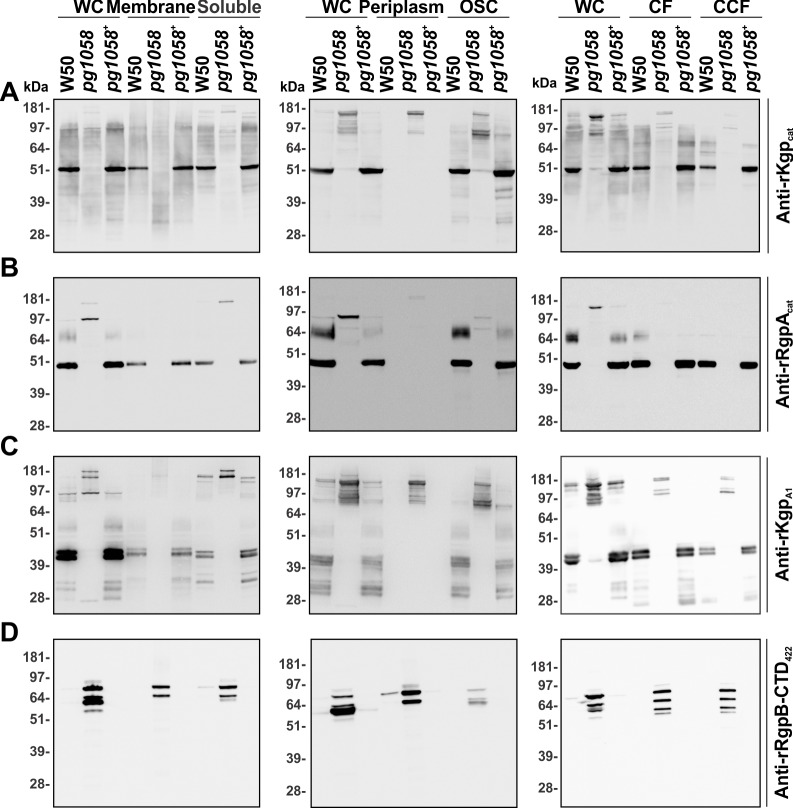
Aberrant localisation of proteinases in subcellular fractions of the *pg1058* mutant. *P*. *gingivalis* W50, *pg1058* mutant and *pg1058*^*+*^ complement strain cultures were fractionated, separated via SDS-PAGE and immunoblotted on nitrocellulose membranes. The immunoblots have material derived from whole-cells (WC; 2 x 10^8^ cells), total membrane (3 x 10^8^ cells) and soluble (1 x 10^8^ cells) fractions; WC, periplasm and osmotically-shocked cell (OSC) fractions (5 x 10^8^ cells each); WC (2 x 10^8^ cells), culture fluid (CF; from culture containing 2 x 10^9^ cells) and vesicle-free cleared culture fluid (CCF; from culture containing 2 x 10^9^ cells). Immunoblots were probed with either anti-rKgp_cat_ (**A**, 1/200,000 dilution), pre-adsorbed anti-rRgpA_cat_ (**B**, 1/10,000 dilution), anti-rKgp_A1_ (**C**, 1/10,000 dilution) or anti-rRgpB-CTD_422_ (**D**, 1/200,000 dilution) followed by goat anti-rabbit (**A**) or horse anti-mouse (**B**, **C** and **D**) IgG-conjugated HRP secondary antibodies (1/3,000 dilution).

To further interrogate the localisation and processing of substrates in the *pg1058* mutant, W50, *pg1058* mutant and *pg1058*^*+*^ complement strain cultures were separated into subcellular fractions and subjected to immunoblot analyses. Use of antiserum against the Kgp catalytic domain (anti-rKgp_cat_) revealed Kgp as a distinct band of ~51 kDa, corresponding to the mature catalytic domain (Kgp_cat_), in all W50 and *pg1058*^*+*^ complement fractions except for the periplasm fractions where it was absent. Kgp_cat_ was mainly cell-associated in the *pg1058* mutant and detected as a high MW band of ~180 kDa corresponding to the Kgp precursor polypeptide (186 kDa) (**Fig 5A**).

Similar to the Kgp_cat_ result, immunoblot with antiserum against the RgpA catalytic domain (anti-rRgpA_cat_) detected a high MW band (~180 kDa) in the *pg1058* mutant whole-cell, soluble and periplasm fractions, corresponding to the RgpA precursor polypeptide (183 kDa). *P*. *gingivalis* W50 and *pg1058*^*+*^ complement strains possessed a distinct band at ~45 kDa, corresponding to the mature catalytic domain (RgpA_cat_). A series of bands can also be seen in W50 and the *pg1058*^*+*^ complement fractions (~60–80 kDa) which correspond to the MW of the mature post-translationally modified RgpB. The high MW band (~97 kDa) visualised in the *pg1058* mutant is likely to be a partially cleaved precursor form of RgpA (**Fig 5B**).

The Kgp precursor polypeptide consists of the Kgp catalytic domain followed by five adhesin domains (Kgp_A1_, Kgp_A2_, Kgp_A3_, Kgp_A4_ and Kgp_A5_). Similarly, the RgpA precursor polypeptide consists of the RgpA catalytic domain followed by four adhesin domains (RgpA_A1_, RgpA_A2_, RgpA_A3_ and RgpA_A4_). The precursors are both proteolytically processed to each produce a catalytic domain which is non-covalently associated with the cleaved adhesin domains in a RgpA/Kgp complex [57]. The anti-rKgp_A1_ antiserum has been shown to recognise Kgp_A1_ as well as RgpA/Kgp complex proteins with MWs which correspond to the RgpA_A1_, RgpA_A3_, Kgp_A3_ and HagA_A3_ adhesins [58]. Immunoblot with anti-rKgp_A1_ antiserum showed high MW bands (~170–190 kDa) in all fractions of the *pg1058* mutant, which corresponded to the MW of Kgp, RgpA and HagA full-length and partially processed precursors. W50 and the *pg1058*^*+*^ complement possessed bands of ~41 kDa and ~43 kDa in all fractions except for the periplasm, which based on identification of bands in Glew *et al*. [34] correspond to fully processed Kgp_A1_ and RgpA_A1_. Proteins with MWs corresponding to processed and modified RgpA_A4_ (45–55 kDa) and RgpA and Kgp polypeptide precursors (97–181 kDa) were also detected in W50 and the *pg1058*^*+*^ complement (**Fig 5C**).

Using antiserum raised against the CTD sequence of RgpB (anti-rRgpB-CTD_422_) showed that the *pg1058* mutant possessed abundant RgpB CTD in all fractions. Due to sequence conservation within the CTD of the CTD-family proteins [15], the anti-rRgpB-CTD_422_ antiserum may cross-react with the CTDs of other T9SS substrates. Protein bands were detected that ranged between ~51 and 181 kDa, that may include RgpB, RgpA and Kgp precursors (78, 183 and 186 kDa respectively). In contrast, *P*. *gingivalis* W50 and the *pg1058*^*+*^ complement possessed minimal CTD-family proteins with which the anti-rRgpB-CTD_422_ antiserum reacted. Overall this suggests that in the *pg1058* mutant the CTD is not proteolytically cleaved from RgpB and other T9SS substrates as they are in the W50 wild-type. The RgpB CTD was detected in both the culture fluid (CF) and vesicle-free cleared culture fluid (CCF) with equivalent signal intensity suggesting the RgpB precursor was soluble and not vesicle-associated (**Fig 5D**).

**Fig 6:**
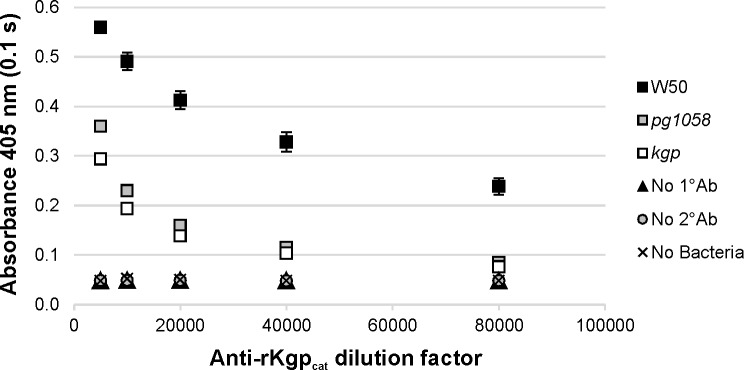
Absence of surface-associated Kgp in the *pg1058* mutant determined by whole-cell ELISA analysis. FKWC (10^9^ per well) prepared from *P*. *gingivalis* W50 (positive control), *pg1058* mutant and *kgp* mutant (negative control) were probed with two-fold dilutions of anti-rKgp_cat_ antisera (1/5,000–1/80,000) followed by goat anti-rabbit HRP-conjugated IgG (1/3,000) and developed with ABTS chromogenic substrate. The chromogenic reaction was stopped and detected at 405 nm. Controls included no primary antibody control (No 1°Ab), no secondary antibody control (No 2°Ab) and no cell control (No Bacteria). Mean ± SEM (standard error of the mean), *N* = 3 for each strain.

Enzyme-linked immunosorbent assay (ELISA) using formalin-killed whole-cells (FKWC) and anti-rKgp_cat_ antisera showed that at antisera dilutions of 1/10,000-1/80,000 there was no significant difference between the *pg1058* and *kgp* mutant strains in the level of Kgp_cat_ detected (*p*<0.05), which was significantly lower than that detected for W50 at each dilution (*p*<0.05). This demonstrates the absence of Kgp on the surface of the *pg1058* mutant (**Fig 6**).

Combined these data confirm that T9SS substrates including RgpA, RgpB and Kgp are aberrantly translocated in the *pg1058* mutant compared to W50, being found predominantly in the periplasm as precursor forms rather than OM-associated mature forms.

### Presence of A-LPS in the *pg1058* mutant

**Fig 7:**
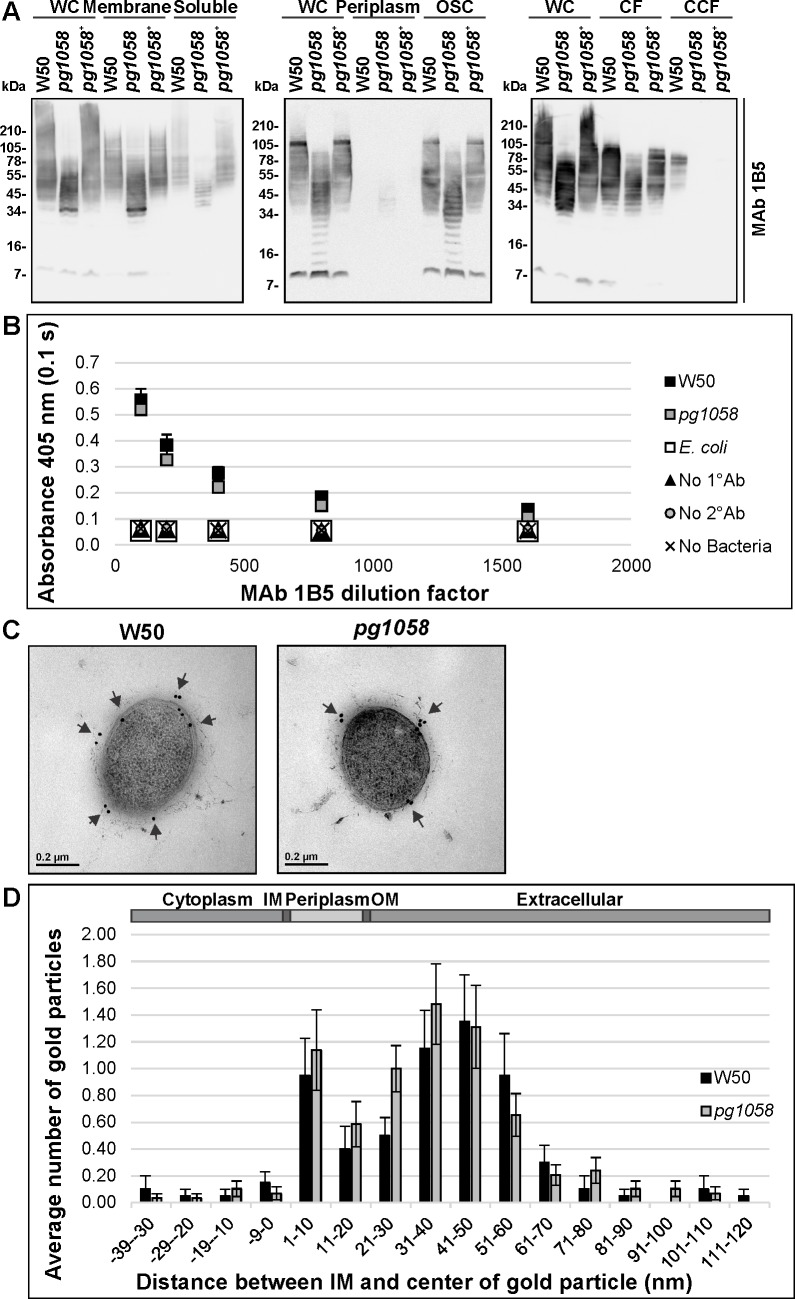
Presence of A-LPS in the *pg1058* mutant. **A.** *P*. *gingivalis* W50, *pg105*8 mutant and *pg1058*^*+*^ complement strain cultures were fractionated, separated via SDS-PAGE and immunoblotted on nitrocellulose membranes (as per Fig 5). Immunoblots were probed with MAb 1B5 (1/10,000 dilution) followed by horse anti-mouse IgG-conjugated HRP secondary antibody (1/3,000 dilution). **B.** Whole-cell ELISA of *P*. *gingivalis* W50 and *pg1058* mutant FKWC (10^9^ per well) probed with two-fold dilutions of MAb 1B5 from 1/100–1/1,600 followed by horse anti-mouse HRP-conjugated IgG secondary antibody (1/3,000) and developed with ABTS chromogenic substrate. The chromogenic reaction was stopped and detected at 405 nm. Controls included no primary antibody control (No 1°Ab), no secondary antibody control (No 2°Ab) and no cell control (No Bacteria). Mean ± SEM, *N* = 3 for each strain. **C.** Immunogold TEM micrographs representative of the *P*. *gingivalis* W50 (*N* = 19) and *pg1058* mutant (*N* = 29). Probing with MAb 1B5 (1/10,000 dilution) was followed by detection with anti-mouse IgG conjugated to 18 nm colloidal gold particles (1/40 dilution). **D.** The immunogold particle distribution. Distance between IM and centre of the gold particle measured in nm, distances less than 0 nm correspond to a cytoplasmic and IM localisation, 0–20 nm corresponds to the periplasmic space, 21–120 corresponds to the OM and extracellular environment. Mean ± SEM.

T9SS substrates are post-translationally modified with A-LPS, which is proposed to anchor the proteins to the cell surface [21, 33, 34]. Given that the T9SS substrates were aberrantly accumulating in the *pg1058* mutant periplasm, analyses were performed to determine if A-LPS was present in the mutant (**Fig 7**). Immunoblot of subcellular fractions from the W50, *pg1058* mutant and *pg1058*^*+*^ complement strains using MAb 1B5, an APS-specific antibody, recognised material in all fractions except for the periplasm. High MW material (~34–105 kDa) was present in W50 and the *pg1058*^*+*^ complement strains, whilst the *pg1058* mutant possessed lower MW material of mostly ~7–78 kDa. The material was more strongly associated with the membrane and osmotically-shocked cell fractions of all strains, and was also likely to be vesicle-associated due to its reduced signal in the CCF. The comparatively low MW of the cell-associated material in the *pg1058* mutant relative to W50 suggests that in this mutant the A-LPS is not conjugated to the T9SS substrate proteins (**Fig 7A**).

The presence of A-LPS on the surface of the *pg1058* mutant was indicated by whole-cell ELISA which showed there was no significant difference in the level of surface-associated A-LPS detected between W50 and the *pg1058* mutant at the antisera dilutions tested (*p*>0.05), which were both significantly greater than that detected for the *E*. *coli* negative control strain which does not produce A-LPS (*p*<0.05) (**Fig 7B**). This was confirmed by immunogold TEM using MAb 1B5 whereby the distribution of gold particles relative to the IM suggested that the APS component of A-LPS recognised by MAb 1B5 is associated with the cell surface and extracellular environment in both W50 and the *pg1058* mutant (**Fig 7C and 7D**).

Combined, these results indicated that while T9SS substrates were not secreted, processed or surface-associated in the *pg1058* mutant as they were in W50, A-LPS was produced, secreted and surface-associated indicating that PG1058 is not essential for A-LPS secretion.

### PG1058 is not essential for maintenance of OM integrity

Proteins with OmpA_C-like domains have been proposed to stabilise the cellular architecture by providing a connection between the OM and the peptidoglycan layer [59]. It was therefore considered prudent to explore the possibility that the T9SS defect observed in the *pg1058* mutant could stem from perturbed incorporation of the T9SS apparatus into the OM due to aberrant cellular architecture (beyond the loss of EDSL). Gram stain of the wild type and *pg1058* cells showed that both were coccoid, with no evidence of elongation or distorted morphology in the *pg1058* mutant (*data not shown*). TEM showed that the *pg1058* mutant possessed normal OM architecture when compared to W50 with no gross difference in the appearance of the OM, peptidoglycan layer or division septa and no evidence of OM detachment or blebbing at the cell poles (**S3A Fig**). Antimicrobial sensitivity disc diffusion assays using chloramphenicol, metronidazole, tetracycline, SDS and Triton X-100 also indicated no notable difference in susceptibility between mutant and W50 (**S3B Fig**), based on current standards which specify a difference in zones of inhibition of >4 mm between susceptible and resistant strains [60]. These data indicate that the gross cell architecture is not compromised and the OM permeability barrier is intact in the *pg1058* mutant.

### PG1058 is an OM-associated periplasmic protein

**Fig 8:**
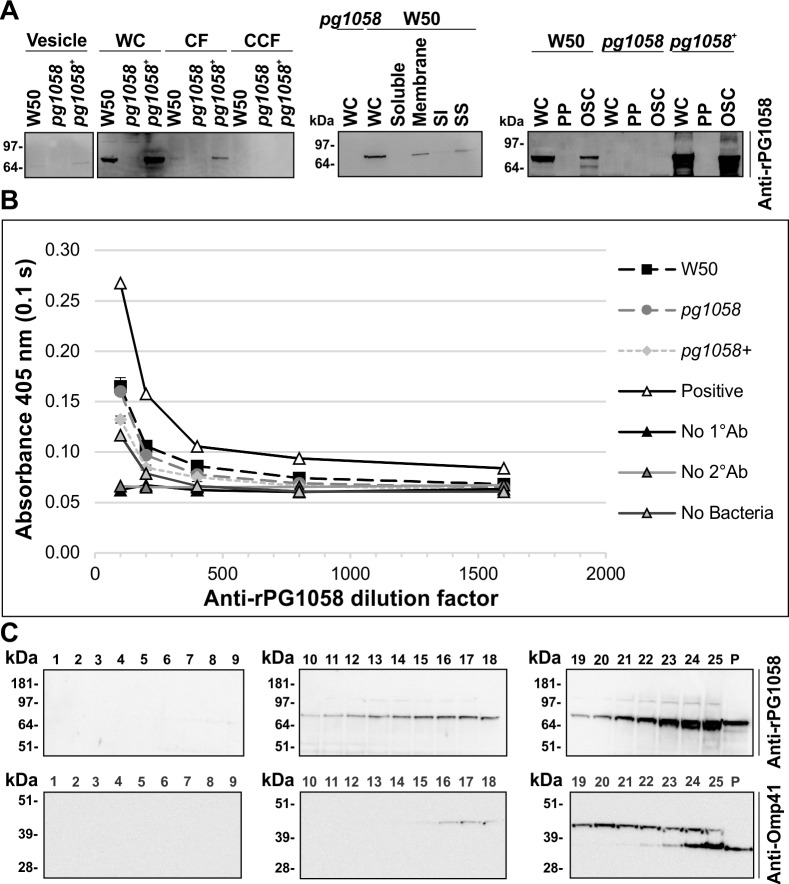
PG1058 is an OM-associated periplasmic protein. **A.** *P*. *gingivalis* W50, *pg1058* mutant and *pg1058*^*+*^ complement strain cultures were fractionated, separated via SDS-PAGE and immunoblotted on nitrocellulose membranes. The immunoblots have material derived from vesicles (5 x 10^9^ cells); whole-cells (WC; 2 x 10^8^ cells), culture fluid (CF; from culture containing 2 x 10^9^ cells) and vesicle-free cleared culture fluid (CCF; from culture containing 2 x 10^9^ cells); WC (2 x 10^8^ cells), soluble (5 x 10^9^ cells), total membrane (Membrane; 1.6 x 10^10^ cells), sarkosyl-insoluble membrane (SI; 1.6 x 10^10^ cells) and sarkosyl-soluble membrane (SS; 1.6 x 10^10^ cells) fractions; WC (5 x 10^8^ cells), periplasm (5 x 10^8^ cells) and osmotically-shocked cell (OSC; 5 x 10^8^ cells) fractions. Immunoblots were probed with anti-rPG1058 (1/10,000 dilution) and horse anti-mouse IgG-conjugated HRP secondary antibody (1/3,000 dilution). **B.** Whole-cell ELISA. *P*. *gingivalis* W50, *pg1058* mutant, *pg1058*^*+*^ complement FKWC or lysed *P*. *gingivalis* W50 FKWC as a positive control (2 x 10^9^ cells per well) were probed with anti-rPG1058 antiserum (1/100, 1/200, 1/400, 1/800 and 1/1600 dilutions) followed by horse anti-mouse HRP-conjugated IgG secondary antibody (1/3,000) and developed with ABTS chromogenic substrate. The chromogenic reaction was suspended and detected at 405 nm. Other controls included no primary antibody control (No 1°Ab), no secondary antibody control (No 2°Ab) and no cell control (No Bacteria). Mean ± SEM, *N* = 3 for each strain except where only one replicate (*N* = 1) of the positive control (Positive) was assessed. **C.** *P*. *gingivalis* W50 total membrane was separated via sucrose density gradient centrifugation then fractions were separated by SDS-PAGE and immunoblotted on nitrocellulose membrane. Fraction numbers are indicated above the lanes and P refers to the pellet (unequal loading). Immunoblots were probed with anti-rPG1058 (1/10,000 dilution) or anti-Omp41 (1/10,000 dilution) followed by horse anti-mouse IgG-conjugated HRP secondary antibody (1/3,000 dilution).

Immunoblot of subcellular fractions using anti-rPG1058 antiserum indicated PG1058 to be ~75 kDa protein associated principally with the whole-cells of W50 and the *pg1058*^*+*^ complement (**Fig 8A**). This agrees with the predicted MW of 73.0 kDa. PG1058 was also detected in the CF and vesicle fractions, suggesting that PG1058 detected in the CF was due to the presence of low abundance PG1058 in the vesicles. Isolation and differential detergent solubilisation of the total membrane fraction showed that PG1058 associated predominantly with the total membrane fraction and the sarkosyl-soluble membrane fraction, indicating that PG1058 is predominantly in the IM or the inner leaflet of the OM (**Fig 8A**). In addition, PG1058 was found associated with the osmotically-shocked membrane fractions with minimal PG1058 visualised within the soluble periplasmic fraction, which is consistent with PG1058 being membrane-associated.

To investigate whether PG1058 was surface-exposed in *P*. *gingivalis*, a whole-cell ELISA was performed using anti-rPG1058 antiserum. There was little difference in the signal detected between the W50 and *pg1058* mutant whole-cells (*p*>0.05) whereas the signal was enhanced when lysed W50 cells were used to coat the wells (**Fig 8B**). This suggests that *P*. *gingivalis* W50 does not possess surface-exposed PG1058 epitopes, indicating instead that PG1058 has an intraperiplasm location.

Isopycnic sucrose density gradient centrifugation was employed to separate the *P*. *gingivalis* IM and OM from the total membrane fraction. Proteomic analyses of fractions 16–25 showed that the membrane proteins were predominantly found in fractions 21–25. IM proteins were most abundant in fractions 21–23 while the OM proteins were clearly enriched in fractions 24 and 25, indicating effective separation of the IM and OM (**S4 Fig**). Immunoblot using anti-rPG1058 showed that PG1058 was broadly dispersed, being detectable in fractions 10–25 as well as the pellet, but was most abundant in fraction 25 (**Fig 8C**). Immunoblot analysis of the integral outer membrane Omp41 showed the high MW form of Omp41 in fractions 15–25 with a low MW form found in fractions 21–25 as well as the pellet (**Fig 8C**). The most intense Omp41-specific signal was in fraction 25, suggesting co-localisation of PG1058 and Omp41 in the same cell fraction. This again suggests that PG1058 is associated with the OM. Together the data indicate that PG1058 is present in the periplasm of *P*. *gingivalis* and is anchored to the inner leaflet of the OM.

## Discussion

The T9SS of the Bacteroidetes and Chlorobi phyla is the most recently identified of the bacterial protein secretion systems. The T9SS is involved in the OM secretion of proteins, which are subsequently surface-associated in some species through glycosyl modification [15, 17, 20, 28]. A T9SS defect can result from disruptions to any of the many genes coding for components of the linked secretion, A-LPS synthesis and modification processes. Prior to the study presented here, more than twenty *P*. *gingivalis* proteins have been shown to affect protein secretion and glycosylation, particularly of the gingipains. The bioinformatic approach employed herein identified twenty-nine proteins as putative T9SS components, sixteen of which were shown experimentally to affect T9SS substrates and three which were previously identified by a bioinformatic investigation alone [27]. Thus ten new candidate T9SS components have been identified, most with putative broad functional assignments or no predicted function (**Table 1**).

In this study we chose to test the hypothesis that PG1058 is a T9SS component. PG1058 was initially identified as a protein (PG4) with sequence homology to OmpA_C of *E*. *coli*, during a screen for potential vaccine antigen targets produced by *P*. *gingivalis* W50 [39]. In the *P*. *gingivalis* W83 genome sequence deposited at NCBI, *pg1058* was annotated as coding for an OmpA-family protein [61]. OmpA is not a known component of a protein secretion system so the annotation may be one reason PG1058 was overlooked in earlier searches for T9SS components.

This study demonstrated that *pg1058* is the third gene in an operon that includes *pg1056* and *pg1057*, which suggested that the proteins coded by the operon may be functionally associated. Homology searching suggests that *pg1056* codes for a putative 6-pyruvoyl tetrahydrobiopterin synthase (QueD) whilst *pg1057* codes for a putative 7-carboxy-7-deazaguanine synthase (QueE). These enzymes are involved in the synthesis of queuosine (nucleoside Q), a modified guanosine derivative found in some tRNAs [62]. Several genes involved in the queuosine biosynthesis pathway form a *queCDEF* operon in a number of species including *Bacillus subtilis* and *Acinetobacter* spp. [63]. There is no similarity between *pg1058* and *queC*, *queF* or any other genes involved in queuosine biosynthesis. In *P*. *gingivalis* W83, *queC* and *queF* homologues are likely to be *pg1310* and *pg1347* respectively. Although the *pg1056*-*pg1058* gene arrangement is conserved within some species of the Bacteroidetes, it is not ubiquitous. The association of *pg1056* and *pg1057* with *pg1058* is likely coincidental and due to a recombination event, frequently observed in *P*. *gingivalis* [64].

Cleavage of the CTD, glycosyl modification and surface-association of the T9SS substrates is a feature common to the T9SS of several Bacteroidetes spp., with A-LPS suggested to be the anchor in *P*. *gingivalis* [20, 21, 33, 34, 65]. In this study, inactivation of *pg1058* resulted in a loss of cell-associated proteinase activity and immunoblotting indicated that Kgp, RgpA and RgpB precursor proteins accumulated in the periplasm of the *pg1058* mutant. This was confirmed by proteomic analyses which showed these and other T9SS substrates were accumulating in the mutant’s periplasm, which coincided with the depletion of T9SS substrates in the membrane fraction. Combined this provided strong evidence that PG1058 is a component of the T9SS in *P*. *gingivalis* essential for the efficient secretion of the T9SS substrates.

Immunoblotting of *P*. *gingivalis* subcellular fractions with an APS-specific antibody (MAb 1B5) suggested that A-LPS was present, however the lower MW indicated it was not conjugated to the T9SS substrates. It was subsequently confirmed that A-LPS was present on the surface of the *pg1058* mutant, with abundance similar to that of W50. Together this suggested that the A-LPS involved in the modification and surface-association of the T9SS substrates in *P*. *gingivalis* can be transported independently of the T9SS.

A non-capsule electron-dense surface layer (EDSL) is present on the surface of *P*. *gingivalis* cells [33]. Mutants with defective secretion of CTD-family proteins (*porT*, *porV*, *porU*) as well as mutants lacking A-LPS (*porR*, *wbaP*) have no EDSL [22, 23, 33, 34, 66, 67] suggesting involvement of both T9SS substrates and A-LPS in EDSL formation. A triple gingipain mutant was shown to have considerably reduced EDSL supporting the presence of gingipains in this layer but also suggesting the involvement of other T9SS substrates and/or A-LPS [23]. The involvement of T9SS substrate proteins in the electron density was confirmed in this study with the *pg1058* mutant that lacks surface-associated T9SS substrates (**Fig 6**) shown to be devoid of EDSL (**Fig 3D**). The presence of surface-associated A-LPS on the *pg1058* mutant indicates that A-LPS alone is not sufficient to generate a detectable level of electron density (**Fig 7B–7D**). Therefore the absence of EDSL in A-LPS mutants is likely due to CTD-family proteins (T9SS substrates) not being surface-associated.

It was considered that the T9SS defect may have resulted from perturbation of the OM and peptidoglycan architecture of the cell since PG1058 possessed sequence homology to TolB and Pal, proteins involved in maintenance of OM integrity. However, antimicrobial sensitivity of the *pg1058* mutant was similar to W50, which indicated an intact OM permeability barrier (**S3B Fig**). Furthermore, TEM indicated that the morphology and architecture of W50 and the mutant were similar (**S3A Fig)**. In contrast, *tol* and *pal* depletion mutants of Gram-negative *Caulobacter crescentus* and *Erwinia chrysanthemi* exhibited membrane blebbing and abnormal division septa [68, 69]. Furthermore, *P*. *gingivalis* ATCC 33277 *pgm6*/*pgm7* deletion mutants lacking OmpA homologues had irregular, wavy OM architecture and increased proteinase activity [70]. Thus it was concluded that PG1058 does not play a crucial role in maintenance of OM integrity.

PG1058 was predicted to be a lipoprotein in *P*. *gingivalis* thus localised to the IM or OM through N-terminal acylation. In this study, analysis of osmotically-shocked *P*. *gingivalis* cells demonstrated the presence of PG1058 in the residual osmotically-shocked membrane fraction but not the soluble periplasm fraction, confirming that PG1058 is anchored to the cell structure (**Fig 8A**). The vesicles and the sarkosyl-soluble fraction of the *P*. *gingivalis* total membrane were shown to harbour the majority of PG1058 present in the cell (**Fig 8A**). It has been proposed that the sarkosyl-soluble fraction consists of the phospholipids of the IM as well as the inner leaflet of the OM [71, 72]. The presence of PG1058 in vesicles as well as the sarkosyl-soluble membrane fraction suggests that PG1058 localises to the inner leaflet of the OM, since vesicles prepared from *P*. *gingivalis* lack IM proteins [73]. The OM localisation of PG1058 is further supported by the co-localisation of integral outer membrane Omp41 with PG1058 in membrane fractions separated by isopycnic sucrose density gradient centrifugation, enriched for OMPs (**Fig 8C**). A whole-cell ELISA indicated that PG1058 is not surface exposed in *P*. *gingivalis* supporting a periplasmic localisation (**Fig 8B**). Structural prediction of PG1058 also suggested that the C-terminal domain is similar to peptidoglycan binding proteins in other bacteria. This prediction would be consistent with PG1058 bridging between the OM and peptidoglycan from N to C terminus.

In all known two-step secretion systems utilising Sec for IM transit, there is a mechanism by which the substrates transit the periplasm. This may be through interaction with a chaperone (type VIII and chaperone/usher pathway) or through interaction with a periplasm spanning complex (type II) [74–76]. Therefore it is expected that the T9SS substrates will interact with T9SS components within the periplasm. Of the sixteen proteins shown experimentally to influence T9SS secretion, fourteen have been specifically localised; four to the IM (PorL, PorM, PorX and PorY), eight to the OM (LptO, PorK, PorN, PorP, PorQ, PorT, Sov and PG0534) and two as OM-associated extracellular proteins (PorU and PG1604) [27, 31, 33, 34, 38, 73, 77, 78]. PG1058 is the first of the T9SS components to have been localised to the periplasm via anchoring to the inner leaflet of the OM.

The most N-terminal domain of PG1058 contains three TPR repeats. TPR repeats form two antiparallel α–helices and are commonly found as tandem arrays of 3–16 copies, however individual or several blocks of TPR motifs can be dispersed among the protein sequence [40, 41]. A right-handed superhelical structure can form from multiple TPR copies, which has a concave groove for interaction with a protein ligand. The TPR motif is conserved based on residues of structural importance where bulky aromatic residues such as tyrosine and phenylalanine juxtapose small residues such as glycine and alanine, rather than specific functional residues. The “hypervariable” residues dictate the specific ligand interaction [41, 79]. Within the hypervariable region of the PG1058 homologues, lysine residues are particularly well conserved, as are aspartic acid and glutamic acid, suggestive of a possibly charged surface for ligand binding (**Fig 2**).

The WD40 repeats of β–propellers are canonically 40–60 residues and end with a conserved tryptophan-aspartate (WD) dipeptide [42]. Each blade of the propeller is formed by a single WD40 repeat folded into a four-stranded antiparallel β-sheet [42, 43]. The β-propeller domain also exhibits residue conservation based on structural constraints on the topology [33]. The residues which are well conserved between blades would likely be of structural relevance, with the small glycine and aspartic acid residues conserved in the PG1058 homologues possibly aiding the packing of blades (**Fig 2**). As with the TPR domain, hypervariable residues of PG1058 would be those most likely to be relevant for specific function. TPR domains are involved in protein-protein interactions and can form large homomultimers or act as scaffolds in multiprotein complexes [41, 47, 79]. Similarly β–propeller proteins are structurally rigid and can form scaffolds for larger protein complexes [43, 80]. It is therefore conceivable that the TPR and β-propeller domains of PG1058 could serve as a structural scaffold for assembly of T9SS components.

The LSX_2_RAX_2_VX_3_L motif is conserved in the family of OmpA_C-like peptidoglycan binding domains [44, 46] and is also present in PG1058 and its homologues (**Fig 2**). The N-terminal domains of proteins with OmpA_C-like domains have variable topologies. Integral OMPs such as OmpA have OM β–barrel domains [81], lipoproteins such as Pal have short, lipidated N-terminal sequences for anchorage to the OM [82] and MotB has a trans-membrane helix for anchorage to the IM [46]. Others such as RmpM of *N*. *meningitidis*, have undetermined N-terminal domain structures [45]. Structural modelling of *P*. *gingivalis* PG1058 against RmpM suggests it has residues in the region where RmpM has an extended alpha helix, followed by an additional helix not present in the *H*. *pylori* MotB and *H*. *influenzae* Pal proteins [45] (**Fig 1**). PG1058 would likely also have structural features in this region not evident in the other proteins. Overall, the structure of PG1058 is predicted to be far more complex than that of other OmpA-family proteins, thus PG1058 and its homologues may represent a new group of peptidoglycan binding proteins.

The CRD fold was originally identified as a non-catalytic β-barrel or β-sandwich fold in the duck hormone processing regulatory enzyme carboxypeptidase D [83], although the function of this domain has not yet been experimentally shown. In the PG1058 homologues, the CRD domain demonstrates the most sequence variability, in both composition and length.

The intricacy of substrate secretion and modification, and the potential number of T9SS components indicates that the T9SS may have a complexity that is yet to be fully appreciated. Further investigation of the newly identified T9SS component PG1058 is warranted in order to elucidate its precise function.

Using comparative genome sequence analysis we identified ten novel candidate components of the T9SS in *P*. *gingivalis* and other Bacteroidetes. One of these, PG1058, was shown in this study to be essential for function of the T9SS in *P*. *gingivalis*. PG1058 is a periplasmic protein anchored to the OM, and appears to be a multidomain protein of novel structure.

## Materials and Methods

### Bioinformatic prediction of T9SS components

A reference protein data set was produced for identification of T9SS candidate proteins. The dataset comprised the entire proteome of selected Bacteroidetes spp. containing predicted T9SS substrates, labelled “CTD-positive” and species from Bacteroidetes and other phyla that did not have predicted T9SS substrates, labelled “CTD-negative” [73]. *P*. *gingivalis* W83 was selected as the reference proteome for comparison. Bacteroidetes CTD-positive species were *Capnocytophaga ochracea* DSM 7271, *Chitinophaga pinensis* DSM 2588, *C*. *hutchinsonii* ATCC 33406, *Dyadobacter fermentans* DSM 18053, *Flavobacteriaceae Bacterium* 3519 10, *F*. *johnsoniae* UW101, *Flavobacterium psychrophilum* JIP02, *Gramella forsetii* KT0803, *P*. *distasonis* ATCC 8503, *Pedobacter heparinus* DSM 2366, *P*. *gingivalis* W83, *R*. *marinus* DSM 4252, *Robiginitalea biformata* HTCC2501, *Salinibacter ruber* DSM 1385 and *Spirosoma linguale* DSM 74, downloaded from NCBI, plus *Prevotella intermedia* 17 and *T*. *forsythia* ATCC 43037 obtained from the ORALGEN database (www.oralgen.org, no longer supported). Bacteroidetes CTD-negative species were *Bacteroides fragilis* NCTC 9343, *B*. *thetaiotaomicron* VPI-5482 and *Bacteroides vulgatus* ATCC 8482. An additional five non-Bacteroidetes spp. were also used as CTD-negative species. These were *Clostridium tetani* E88, *E*. *coli* K102, *Neisseria gonorrhoeae* FA 1090, *N*. *meningitidis* MC58 and *Pseudomonas aeruginosa* PA7, downloaded from NCBI. The differential analysis used BLASTp [84] where each protein sequence in *P*. *gingivalis* was aligned with each protein sequence in the other Bacteroidetes spp. Default parameters were used during the BLASTp search. The BLASTp score for the best hit for each alignment was obtained and put into a scoring matrix. Batch BLAST was used in order to automate and accelerate the alignments. Three parameters were then used as filters to select protein sequences that were conserved amongst the CTD-positive and absent in the CTD-negative species; mean positives scores (MPS), mean negatives scores (MNS) and ratio of MPS over MNS (Difference Ratio). The parameters were optimised by analysing the parameter values for sequences of proteins shown experimentally to be T9SS components. These were PorK, PorL, PorM, PorN, PorP, PorQ, PorT, PorU, PorV, PorW, PorX, PorY and Sov. The optimised cut-off values were MPS ≥ 80, MNS ≤ 80 and Difference Ratio ≥ 2. The final filter applied was that each *P*. *gingivalis* protein must have a BLASTp score higher than the ANS in at least ten CTD-positive organisms. The list of candidates was then validated by BLASTp searching against the entire NCBI non-redundant protein database using the default parameters. Each candidate was only accepted if the top 100 protein matches were all from the Bacteroidetes phylum.

PG1058 homologues in Bacteroidetes spp. were identified by BLASTp searches using the *P*. *gingivalis* W83 PG1058 as the query. The list of candidate homologues were refined by retaining only those homologues with the lowest expect value for a particular strain, exhibiting more than 60% sequence coverage and containing all four PG1058 domains (TPR, β-propeller, CRD or OmpA_C-like). Sequences that had regions relative to PG1058 of more than 70 additional residues were removed (47 sequences) and examined separately. Multiple sequence alignments were produced using the COBALT (constraint-based alignment tool for multiple protein sequences) [85] and CLUSTAL W [51] tools in BioEdit [86]. A sequence logo indicating sequence conservation was generated from the alignment using WebLogo 3 [52, 53].

### Bacterial growth conditions

*P*. *gingivalis* strains were grown anaerobically (80% N_2_, 10% H_2_, and 10% CO_2_; MG500 anaerobic workstation; Don Whitley Scientific Ltd.) at 37°C in Heart Infusion (HI, Oxide) broth (3.7% w/v) supplemented with 5 μg/mL haemin and 0.5 mg/mL cysteine hydrochloride and maintained on horse blood agar (HBA; 3.7% w/v Blood agar base No. 2, Oxoid; 10% (v/v) lysed horse blood). Bacterial strains used in this study are listed in **S1 Table**.

*P*. *gingivalis* cell numbers were determined by comparing culture OD to a growth curve previously prepared by this laboratory where OD was related to cell numbers. The OD_650_ 0.6 is equivalent to approximately 2.5 x 10^9^ cells/mL for *P*. *gingivalis*, which is within the exponential phase of growth.

### Inactivation and complementation of *pg1058*

*P*. *gingivalis* W50 genomic DNA (gDNA) was isolated using the DNeasy® Blood and Tissue kit (Qiagen). Ligated plasmids were used to transform *E*. *coli* α-Select cells (Bioline) and sequences verified. All oligonucleotide primers used in this study are listed in **S2 Table**. An internal region of *pg1058* (1935 nt) was amplified from *P*. *gingivalis* W50 gDNA using primers PG1058_fwd1 and PG1058_rev1 and ligated into pGEM^®^-T Easy (Promega). A 731 nt *pg1058* fragment was excised from this plasmid by BsaBI digestion and replaced by *ermF* obtained by RsaI digestion of pVA2198 [87]. This suicide plasmid was linearised by PstI digestion and used to transform *P*. *gingivalis* W50 by electroporation [88]. Transformants were selected on HBA plates supplemented with erythromycin (10 μg/mL). Correct homologous recombination into the transformant genome was determined by PCR. A verified transformant with the internal region of *pg1058* deleted and replaced by *ermF* was designated the *pg1058* mutant strain ECR370.

The *pg1058* mutant was complemented *in trans* with a copy of the *pg1058* ORF. The *cepA* gene and promoter was PCR amplified from plasmid pEC474 [89] using primers cepAf and cepAr, ligated into the pGEM^®^-T Easy plasmid, generating pCS19. Primers PG0176ntigrNcoIFWD and PG0176LdrREsREV were used to amplify the DNA upstream of the *P*. *gingivalis* W50 *mfa1* gene with the addition of a 5’ NcoI RE site and a 3’ multiple cloning site. This amplicon was digested with NcoI and SacII, ligated to NcoI/SacII-digested pCS19, generating p*Mfa1*_up. A second amplicon containing the 5’ region of *mfa1* was produced with the addition of a 5’ SpeI and 3’ NdeI RE site using primers PG0176GnSpeIFWD and PG0176GnNdeIREV. This amplicon was SpeI/NdeI-digested, ligated to SpeI/Nde-digested p*Mfa1*_up, generating p*Mfa1cepA* in which *cepA* is flanked 5’ by DNA homologous to the nucleotides upstream of *mfa1* and 3’ by the start of the *mfa1* ORF. The *pg1058* ORF (2,042 nt) was amplified from *P*. *gingivalis* W50 gDNA with the addition of a 5’ BamHI and 3’ SacII RE site using PG1058compBamHIFor and PG1058compSacIIRev primers and ligated into pGEM^®^-T Easy, generating pGEM:*pg1058*. The *pg1058* ORF was excised from pGEM:*pg1058* by digestion with BamHI and SacII and ligated to BamHI/SacII-digested p*Mfa1cepA*, generating p*1058cepA* with *pg1058* in the same orientation as *mfa1*. This recombination cassette was excised by digestion with DraIII and SacI and used to transform the *pg1058* mutant (ECR370). A transformant was selected on HBA supplemented with ampicillin (5 μg/mL), verified and designated the *pg1058*^*+*^ complement strain ECR756.

### *P*. *gingivalis* cell harvesting and fraction preparation

*P*. *gingivalis* cells were harvested from cultures grown to exponential phase by centrifugation at 8,000 g, 4°C for 25 min. During preparation of subcellular fractions, cultures were supplemented with protease inhibitors (1 mM tosyl-L-lysyl-chloromethane hydrochloride, TLCK, Sigma-Aldrich; 1 mM EDTA; 0.1% v/v PIC, Sigma-Aldrich) and additional protease inhibitors (1 mM TLCK, 1 mM EDTA, 0.1% v/v) were included in wash buffers, added to the samples during fractionation and to the final fractions. Harvested cells were washed in TC150 buffer (150 mM NaCl, 50 mM Tris-HCl, pH 8.0) unless indicated otherwise.

Whole-cell extracts designated here as whole-cell fractions (WC), culture fluid (CF) and vesicle-free cleared culture fluid (CCF) fractions were obtained as previously described [33] except for isolation of the CCF by ultracentrifugation of CF at 338,000 *g*, 4°C for 40 min. The vesicle pellets (V) were washed in phosphate buffered saline (PBS, pH 7.4) and retained.

Periplasm fractions (PP) were obtained by osmotic-shock of washed cells as previously described [33] with aliquots of the cell suspensions collected as representative of whole-cell fractions (WC) prior to centrifugation, and pellets retained and designated osmotically-shocked cells (OSC).

Membrane fractions were prepared via differential detergent solubilisation as previously published [16] with minor changes. Samples of washed cells were retained as whole-cell fractions (WC). Cells were lysed using a French^®^ Pressure Cell Press with a 40,000 psi French^®^ Press Cell (SLM Instruments, USA) set to 1,500 psi instead of sonication, and all ultracentrifugation steps were performed at 176,000 *g*, 4°C for 30 min. Supernatants containing soluble cytoplasmic and periplasmic proteins were collected and designated soluble fractions (SOL) and pellets containing the membrane fraction were designated total membrane fractions (Membrane). Separation of the sarkosyl-soluble supernatant (SS) and sarkosyl-insoluble pellets (SI) was performed using a 2% w/v sarkosyl solution instead of 10% w/v, and TC150 buffer was used for all steps instead of PBS.

CF, CCF and PP fractions were precipitated with 12% v/v trichloroacetic acid (TCA), washed with acetone and suspended to 1/10 of the starting volume in 25 mM Tris-HCl (pH 7.5).

The protein concentration of each sample was determined relative to the number of cells used to generate the sample. All protein samples were compared based on equivalent cell numbers.

The *P*. *gingivalis* total membrane was isolated and underwent isopycnic sucrose density gradient centrifugation as previously published [90] with minor changes. Phenylmethylsulfonyl fluoride, leupeptin and RNase were not used in sample preparation; non-lysed cells were removed from cell lysate by centrifugation at 8,000 *g*, 4°C for 25 min; the total membrane was suspended with the aid of sonication on ice for a total of 5 min (pulses of 30 s on, 30 s off) using a CPX750 Ultrasonic processor (20% amplitude) fitted with a 6.5 mm tapered microtip probe (Cole Parmer Instrument Company). The sucrose gradient consisted of 1.7 mL of 2.02 M sucrose, 5.9 mL of 1.44 M sucrose, 4.2mL of 0.77 M sucrose prepared in 10 mM HEPES-NaOH (pH 7.4) in an ultracentrifuge tube (344060, Ultra-Clear™, Beckman Coulter). The relative volume of each sucrose solution was consistent with the published protocol. The gradients were ultracentrifuged at 100,000 *g*, 4°C for 20 h using a SW40Ti rotor (Beckman Coulter™ Inc.). Fractions (0.5 mL) were collected by careful pipetting from the top of the ultracentrifuge tube.

### Immunoblot analysis

*P*. *gingivalis* subcellular fractions were suspended in 1X NuPAGE^®^ LDS Sample Buffer (Invitrogen™) supplemented with 50–100 mM DTT. Samples were resolved by SDS-PAGE and transferred onto nitrocellulose membrane using NuPAGE^®^ reagents and precast gels (Invitrogen™). The membrane was blocked with 5% non-fat skim milk in PBST_0.1_ (0.1% v/v Tween-20 in PBS) and probed with primary antibody followed by a goat anti-rabbit or horse anti-mouse horseradish peroxidase (HRP)-conjugated IgG secondary antibody (Cell Signaling Technology Inc.). The signal was detected by SuperSignal West Pico chemiluminescent substrate (Quantum Scientific) or Immobilon^®^ Western chemiluminescent HRP substrate (Merck Millipore,) and visualised with a LAS-3000 imaging instrument (FUJI). In some cases, the bound antibody was removed using stripping solution (2% w/v SDS; 63 mM Tris-HCl pH 6.8; 0.8% v/v 2-mercapotoethanol, Sigma-Aldrich) and the membranes were then re-probed with different primary and secondary antibodies for detection of different proteins.

Mouse monoclonal antibody 1B5 (MAb 1B5) was a gift from Professor M.A. Curtis, Royal London School of Medicine and Dentistry, London, United Kingdom [19]. Mouse monoclonal antibody anti-Omp41 was a gift from CSL™ (Parkville, Victoria, Australia). Anti-rRgpB-CTD_422_ (previously designated anti-rRgpB-CTD(422)) [34] and anti-rKgp_A1_ [58] were mouse polyclonal antisera raised against purified *E*. *coli* His-tagged recombinant proteins. Anti-rRgpA_cat_ mouse polyclonal antisera was raised against purified *E*. *coli* His-tagged recombinant protein, which was subsequently preadsorbed with *P*. *gingivalis* YH522 *rgpA*^-^, *rgpB*^*-*^ mutant cell lysate [15]. Purified recombinant Kgp enzyme [91] was used to raise anti-rKgp_cat_ rabbit polyclonal antisera. A purified *E*. *coli* His-tagged protein rPG1058 produced using an expression plasmid, which was a gift from CSL Ltd., Australia, was used to raise the mouse polyclonal antisera anti-rPG1058.

### Haemagglutination activity assay

Haemagglutination activity assay was performed using washed *P*. *gingivalis* cells as described previously [33] except for the use of sheep erythorcytes instead of horse.

### Proteinase activity assays

*P*. *gingivalis* cells were washed in TC150 buffer (150 mM NaCl, 50 mM Tris-HCl, 5 mM CaCl_2,_ pH 8.0,) supplemented with cysteine hydrochloride (10 mM), suspended in an equivalent culture volume of the same solution and designated whole-cell fractions. Culture supernatants were ultracentrifuged at 103,900 *g*, 4°C for 40 min and designated supernatant fractions. Fractions were analysed for Arg- and Lys-specific proteinase activities as previously described [91] using chromogenic substrates *N*-α-benzoyl-_L_-Arg-*p*-nitroanilide (BA*p*NA) and Kgp substrate was *N*-Tosylglycyl-_L_-prolyl-_L_-lysine4-nitroanilide acetate salt (L*p*NA). The Arg- and Lys-specific assay mixtures contained either whole-cell fractions (3.8 x 10^7^ and 5 x 10^7^ cells respectively) or supernatant fractions (45 μL and 60 μL respectively), cysteine hydrochloride (10 mM), 2 mM BA*p*NA or L*p*NA and 5% (v/v) dimethyl sulfoxide made up to 200 μL in TC150 buffer. The release of *p*NA was monitored using a microtitre plate reader (Wallac VICTOR™ 3 Multilabel Counter, PerkinElmer™ Pty. Ltd.) by the change in OD at 405 nm over time.

### Cryo-electron microscopy (Cryo-EM)

*P*. *gingivalis* cells were washed in pre-filtered TM20 buffer (20 mM Tris-HCl, pH 7.4, 20 mM NaCl, 10 mM MgCl_2_) and suspended in the same buffer to 1/5 of the original culture volume. Cell suspensions (6 μL) underwent cryo-fixation and TEM imaging under cryogenic conditions as previously described [33].

### Whole-cell enzyme linked immunosorbant assay (ELISA)

*P*. *gingivalis* and *E*. *coli* α-Select cells were harvested from exponential phase cultures and formalin-killed whole-cells (FKWC) were prepared as previously described [33]. Whole-cell ELISAs were performed using FKWC as previously described [33], except where 2 x 10^9^ FKWC were used to coat the wells, the blocking solution contained 5% (w/v) skim-milk in PBST_0.1_ and antibodies prepared in blocking solution were incubated for 1 h. Colour development was stopped via addition of stop solution (final concentration 1 mg/mL NaF) and the optical density was measured at 405 nm (0.1 s) using a microtitre plate reader (Wallac VICTOR™ 3 Multilabel Counter).

### Immunogold transmission electron microscopy

*P*. *gingivalis* cells were washed and suspended in PBS to 1/80 culture volume and incubated on ice. Cell suspensions (2 μL) underwent cryo-fixation and immunogold labelling as previously described [92] using MAb 1B5 (1/10,000 dilution) and anti-mouse secondary antibody conjugated to 18 nm colloidal gold particles (diluted 1:40). Micrographs were taken with a Phillips CM120 Biotwin transmission electron microscope (Koninklijke Philips N.V.) at 120 kV using a Gatan Multiscan 600CW digital camera (Gatan Inc.). Gold particle distribution was enumerated using Image J [93] by measuring the distance between the outer edge of the IM (0 nm) and the centre of the gold particle, rounded to the nearest 5 nm, and comparing the number of gold particles within a given 10 nm region. Gold particles present in the cytoplasm were given negative distances.

### Liquid chromatography tandem mass spectrometry (LC-MS/MS)

For analysis of the entire periplasm fraction (7.5 x 10^9^ cells) or total membrane fraction (4 x 10^8^ cells) samples from a SimplyBlue™ SafeStain stained SDS-PAGE gel, the whole lane was cut into several segments each containing multiple protein bands. Each segment was treated as a separate sample. Proteins in isopycnic sucrose density gradient centrifugation fractions were precipitated using 15% v/v TCA, washed with acetone and suspended in 5.3 M urea in NH_4_HCO_3_. All samples underwent reduction with dithiothreitol, alkylation with iodoacetamide, trypsin digestion and acidification with trifluoroacetic acid as previously described [73]. Peptides underwent LC-MS/MS analysis with the same search parameters as previously published [73]. The subcellular localisation of *P*. *gingivalis* proteins used for analysis of subcellular fractions in this study is summarised in **S3 Table**, based on data generated during preparation of Veith *et al*. [73].

### PG1058 structure prediction

Structure modelling was done using Phyre^2^ [94] with program default parameters. Additional modelling of the β-propeller domain was performed using the InterPro database [95].

### Statistics

Data were analysed using IBM SPSS Statistics Version 22. Data were analysed using a one-way analysis of variance (ANOVA) with a Fisher’s Least Significant Difference (LSD) post hoc test for multiple comparisons with statistical significance at *p*<0.05. Data were assumed to be normally distributed with homogeneous variance.

## Supporting Information

S1 Experimental Procedures (DOCX)

S1 Fig Alignments and secondary structure predictions of PG1058 domains predicted by Phyre^2^. **A.** The TPR domain aligned to 412wA, the myosin chaperone unc-45 from *Caenorhabditis elegans* **B.** The β–propeller domain aligned to c2w8bB, *E*. *coli* TolB. Note the lack of homology in places, which results in fewer blades predicted in PG1058 than in the template molecule. **C.** The CRD domain aligned to cmn8A, *Drosophila melanogaster* carboxypeptidase d isoform 1b2 short. **D.** The OmpA_C domain aligned to c1r1m1, the OmpA-like domain from RmpM of *Neisseria meningitidis*.
(DOCX)

S2 Fig Inactivation and complementation of *pg1058* did not affect the expression of *pg1056* and *pg1057* in the operon. **A.** RT-PCR using a reverse oligonucleotide primer specific for *pg1058* and forward oligonucleotide primers specific for *pg1056*, *pg1057* or *pg1058* transcripts with no template (NTC), W50 gDNA, reverse transcribed W50 RNA and W50 RNA that was not reverse transcribed (No RT) indicated that *pg1058* is the third gene in a three gene operon. **B.** Non-endpoint RT-PCR was performed using oligonucleotide primer pairs specific to *pg1056*, *pg1057* and *pg1058* with no template (NTC), W50 gDNA, reverse transcribed W50 RNA, reverse transcribed *pg1058* mutant RNA and reverse transcribed *pg1058*^*+*^ complement RNA indicated that transcription of *pg1056* and *pg1057* was not affected in the *pg1058* mutant. See S1 Experimental Procedures.
(DOCX)

S3 Fig Normal OM architecture and integrity in the *pg1058* mutant. **A.** TEM analysis of the *P*. *gingivalis* OM architecture. TEM micrographs (93-180k X magnification) representative of the *P*. *gingivalis* W50 and *pg1058* mutant indicate the division septum and cell poles of the *pg1058* mutant appear normal with no evidence of OM blebbing. Outer membrane (OM), inner membrane (IM) and peptidoglycan (PGN), division septum and cell poles (arrows) are indicated. Scale bars are 100 or 200 nm as indicated. **B.** Antimicrobial sensitivity disc diffusion assay. Discs impregnated with chloramphenicol (CHL, 10 μg), metronidazole (MTZ, 1 μg), tetracycline (TET, 0.40 μg), SDS (0.25 μg) or Triton X-100 (0.53 μg) were placed on agar plates seeded with W50 or *pg1058* mutant cells. The zone of growth inhibition (mm) was measured after 24 h. Mean ± SEM; *N* = 4; ** indicates *p*<0.01. See S1 Experimental Procedures.
(DOCX)

S4 Fig Proteomic analyses of isopycnic sucrose density gradient fractionation of the *P*. *gingivalis* total membrane. The combined Mascot score of confirmed non-CTD OM proteins (black bars) and IM proteins (white bars) within each fraction was compared to the total Mascot score for all proteins identified in that fraction as a percentage. *N* = 1.
(DOCX)

S1 Table Bacterial Strains. A, ATCC: American Type Culture Collection. B, Strain ECR669 has *kgp* from the Met codon to the stop codon substituted with *cepA* [89] that codes a cephalosporinase. This mutant was made by flanking *cepA* with >300 nt of DNA immediately 5’ and 3’ to *kgp* and using this to transform *P*. *gingivalis* W50. Replacement of *kgp* occurred after homologous recombination, with transformants selected on HBA supplemented with 5 μg/mL of ampicillin.
(DOCX)

S2 Table Oligonucleotide primers. (DOCX)

S3 Table *P*. *gingivalis* W50 protein localisation used for proteomic analyses. (DOCX)
