# Supplementary material for: PG1058 Is a Novel Multidomain Protein Component of the Bacterial Type IX Secretion System
Source: PLoS One. 2016 Oct 6;11(10):e0164313. doi: 10.1371/journal.pone.0164313 (PMC5053529; doi:10.1371/journal.pone.0164313)
Supplement: S1 Experimental Procedures — (DOCX) [file pone.0164313.s001.docx]

**S1 Experimental Procedures**

**Reverse transcription (RT) PCR**

Total RNA was isolated from *P. gingivalis* using the NucleoSpin^®^ RNA II kit (Macherey-Nagel GmbH & Co KG) with additional DNase treatment using TURBO™ DNase (Invitrogen™). RT was performed using the SuperScript^®^ III Reverse Transcriptase First-Strand Synthesis SuperMix for qRT-PCR kit with random hexamer primers (Invitrogen™). To identify co-transcription, MyTaq™ Red DNA Polymerase (Bioline) was used in a standard PCR as per the manufacturer’s instructions with a reverse oligonucleotide primer specific for *pg1058* (PG1058DomIRev1) and forward oligonucleotide primers specific for *pg1056* (PG1056For1), *pg1057* (PG1057For2) and *pg1058* (PG1058DomIFor1). To semi-quantify transcription of the genes of interest, MyTaq™ DNA Polymerase (Bioline) was used in a non-endpoint PCR of 20 cycles with primer pairs specific to *pg1056* (PG1056For2 and PG1056Rev2), *pg1057* (PG1057For2 and PG1057Rev2) and *pg1058* (PG1058DomIIIFor1 and PG1058DomIIIRev1). Controls were no template, *P. gingivalis* W50 gDNA and RNA that was not reverse transcribed. The PCR products underwent agarose gel electrophoresis in SYBR^®^Safe-stained gels and were imaged using a Las-3000 imaging instrument (Fujifilm).

**Antimicrobial sensitivity disc diffusion assay**

The *P. gingivalis* W50 and *pg1058* mutant were grown to late exponential phase at an OD_650_ of 1.0. Molten trypticase soy agarose (0.8% agarose, Promega, USA; BBL™ Trypticase™ Soy Broth, BD; 10 mL, 55°C) was inoculated with 5 x 10^9^ cells of *P. gingivalis*, which was then overlayed onto a trypticase soy agar plate (1.5% agar, BBL™ Trypticase™ Soy Agar, BD; 5 μg/mL hemin) and allowed to set. Paper discs (5 mm diameter; Whatman^®^ cellulose chromatography paper 3 mm) were placed on top of the medium and impregnated with 5 μL of antimicrobial agent (2 mg/mL chloramphenicol (10 μg), 200 μg/mL metronidazole (1 μg), 80 μg/mL tetracycline (0.40 μg), 5% w/v SDS (0.25 μg), 10% v/v Triton-X 100 (0.53 μg)). Water and ethanol solvent controls, no inoculum and no antimicrobial agent controls were also prepared. Plates were incubated anaerobically at 37°C. The diameter of the clear zone within the bacterial lawn around each disc was measured (mm) after 24 hours.

**Transmission electron microscopy of chemically fixed cells**

*P. gingivalis* were grown to exponential phase, OD_650_ ~0.8. Cells were harvested from culture (35 mL) by centrifugation at 6, 000 *g*, 4°C for 20 min. Cells were washed via suspension in PBS (10 mL) and centrifugation at 6,000 *g*, 4°C for 10 min. Washed cell pellets were suspended in PBS (500 μL) then re-pelleted by centrifugation. Cells were prepared for transmission electron microscopy via chemical fixation as previously described [[1](#_ENREF_1)]. Micrographs were taken as described for immunogold transmission electron microscopy.

**Reference**

1. Hu M, Crawford Simon A, Henstridge Darren C, Ng Ivan HW, Boey Esther JH, Xu Y, et al. p32 protein levels are integral to mitochondrial and endoplasmic reticulum morphology, cell metabolism and survival. Biochemical Journal. 2013;453(3):381-91.
