# Supplementary material for: PG1058 Is a Novel Multidomain Protein Component of the Bacterial Type IX Secretion System
Source: PLoS One. 2016 Oct 6;11(10):e0164313. doi: 10.1371/journal.pone.0164313 (PMC5053529; doi:10.1371/journal.pone.0164313)
Supplement: S1 Fig — A. The TPR domain aligned to 412wA, the myosin chaperone unc-45 from Caenorhabditis elegans B. The β–propeller domain aligned to c2w8bB, E. coli TolB. Note the lack of homology in places, which results in fewer blades predicted in PG1058 than in the template molecule. C. The CRD domain aligned to cmn8A, Drosophila melanogaster carboxypeptidase d isoform 1b2 short. D. The OmpA_C domain aligned to c1r1m1, the OmpA-like domain from RmpM of Neisseria meningitidis. (DOCX) [file pone.0164313.s002.docx]

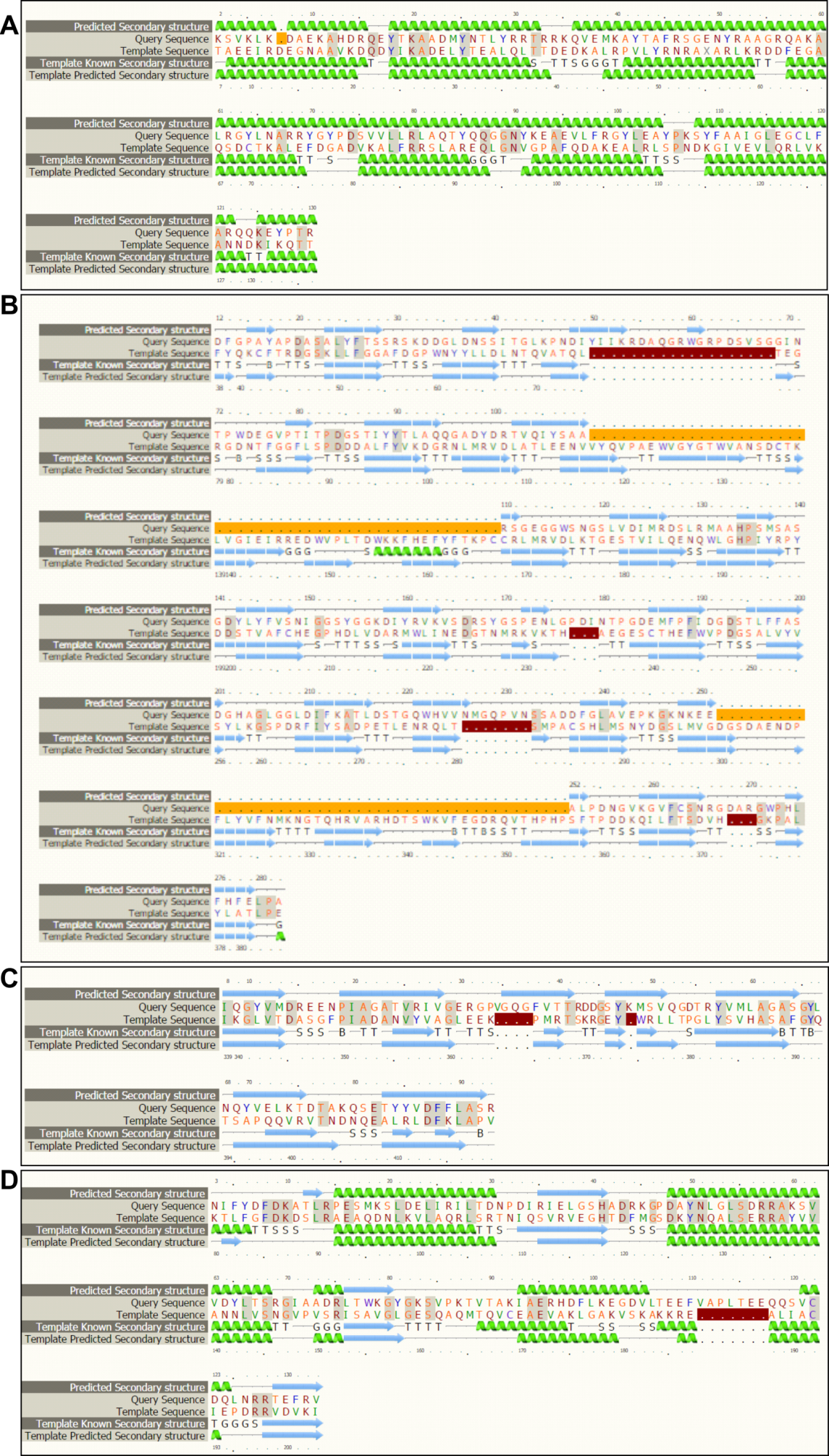


**S1 Fig. Alignments and secondary structure predictions of PG1058 domains predicted by Phyre^2^.** **A.** The TPR domain aligned to 412wA, the myosin chaperone unc-45 from *Caenorhabditis elegans* **B.** The β–propeller domain aligned to c2w8bB, *E. coli* TolB. Note the lack of homology in places, which results in fewer blades predicted in PG1058 than in the template molecule. **C.** The CRD domain aligned to cmn8A, *Drosophila melanogaster* carboxypeptidase d isoform 1b2 short. **D.** The OmpA_C domain aligned to c1r1m1, the OmpA-like domain from RmpM of *Neisseria meningitidis*.
