# Supplementary material for: PG1058 Is a Novel Multidomain Protein Component of the Bacterial Type IX Secretion System
Source: PLoS One. 2016 Oct 6;11(10):e0164313. doi: 10.1371/journal.pone.0164313 (PMC5053529; doi:10.1371/journal.pone.0164313)
Supplement: S2 Fig — A. RT-PCR using a reverse oligonucleotide primer specific for pg1058 and forward oligonucleotide primers specific for pg1056, pg1057 or pg1058 transcripts with no template (NTC), W50 gDNA, reverse transcribed W50 RNA and W50 RNA that was not reverse transcribed (No RT) indicated that pg1058 is the third gene in a three gene operon. B. Non-endpoint RT-PCR was performed using oligonucleotide primer pairs specific to pg1056, pg1057 and pg1058 with no template (NTC), W50 gDNA, reverse transcribed W50 RNA, reverse transcribed pg1058 mutant RNA and reverse transcribed pg1058+ complement RNA indicated that transcription of pg1056 and pg1057 was not affected in the pg1058 mutant. See S1 Experimental Procedures. (DOCX) [file pone.0164313.s003.docx]

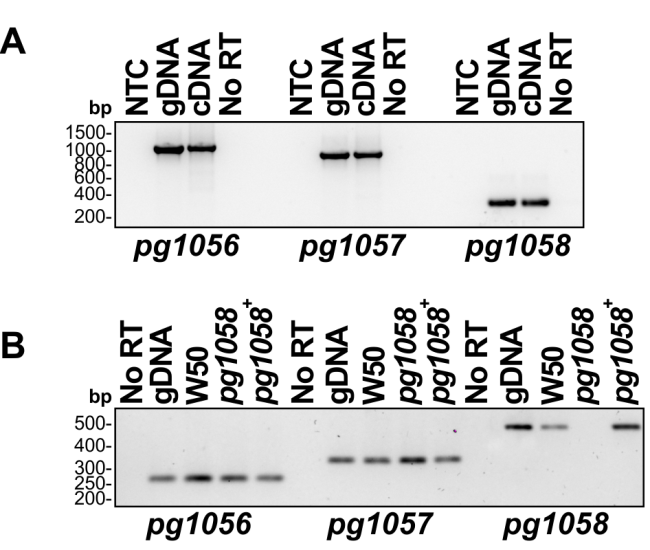


**S2 Fig. Inactivation and complementation of *pg1058* did not affect the expression of *pg1056* and *pg1057* in the operon. A.** RT-PCR using a reverse oligonucleotide primer specific for *pg1058* and forward oligonucleotide primers specific for *pg1056*, *pg1057* or *pg1058* transcripts with no template (NTC), W50 gDNA, reverse transcribed W50 RNA and W50 RNA that was not reverse transcribed (No RT) indicated that *pg1058* is the third gene in a three gene operon. **B.** Non-endpoint RT-PCR was performed using oligonucleotide primer pairs specific to *pg1056*, *pg1057* and *pg1058* with no template (NTC), W50 gDNA, reverse transcribed W50 RNA, reverse transcribed *pg1058* mutant RNA and reverse transcribed *pg1058^+^* complement RNA indicated that transcription of *pg1056* and *pg1057* was not affected in the *pg1058* mutant. See S1 Experimental Procedures.
