# Supplementary material for: PG1058 Is a Novel Multidomain Protein Component of the Bacterial Type IX Secretion System
Source: PLoS One. 2016 Oct 6;11(10):e0164313. doi: 10.1371/journal.pone.0164313 (PMC5053529; doi:10.1371/journal.pone.0164313)
Supplement: S3 Fig — A. TEM analysis of the P. gingivalis OM architecture. TEM micrographs (93-180k X magnification) representative of the P. gingivalis W50 and pg1058 mutant indicate the division septum and cell poles of the pg1058 mutant appear normal with no evidence of OM blebbing. Outer membrane (OM), inner membrane (IM) and peptidoglycan (PGN), division septum and cell poles (arrows) are indicated. Scale bars are 100 or 200 nm as indicated. B. Antimicrobial sensitivity disc diffusion assay. Discs impregnated with chloramphenicol (CHL, 10 μg), metronidazole (MTZ, 1 μg), tetracycline (TET, 0.40 μg), SDS (0.25 μg) or Triton X-100 (0.53 μg) were placed on agar plates seeded with W50 or pg1058 mutant cells. The zone of growth inhibition (mm) was measured after 24 h. Mean ± SEM; N = 4; ** indicates p<0.01. See S1 Experimental Procedures. (DOCX) [file pone.0164313.s004.docx]

**
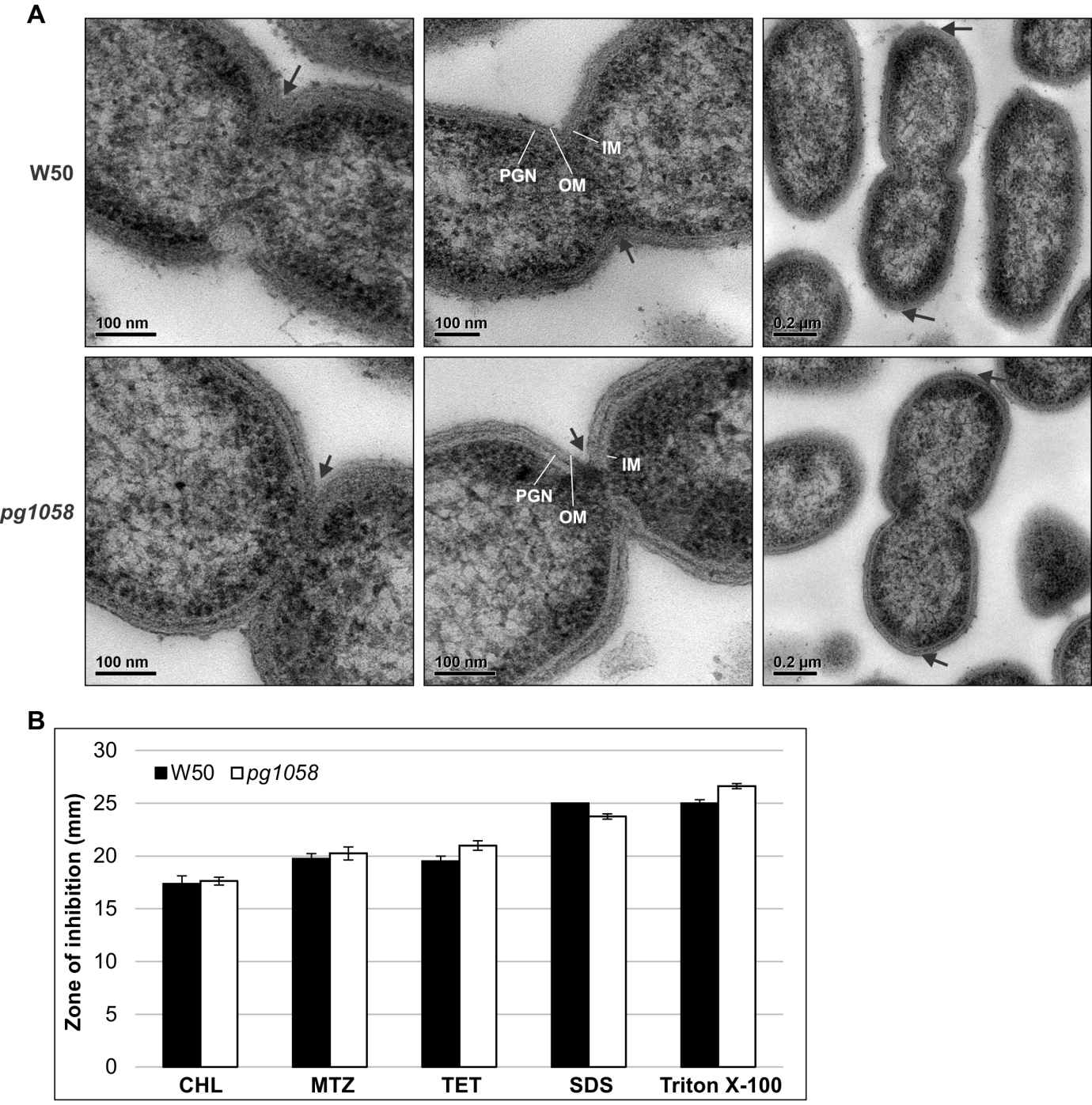
**

**S3 Fig. Normal OM architecture and integrity in the *pg1058* mutant. A.** TEM analysis of the *P. gingivalis* OM architecture. TEM micrographs (93-180k X magnification) representative of the *P. gingivalis* W50 and *pg1058* mutant indicate the division septum and cell poles of the *pg1058* mutant appear normal with no evidence of OM blebbing. Outer membrane (OM), inner membrane (IM) and peptidoglycan (PGN), division septum and cell poles (arrows) are indicated. Scale bars are 100 or 200 nm as indicated. **B.** Antimicrobial sensitivity disc diffusion assay. Discs impregnated with chloramphenicol (CHL, 10 µg), metronidazole (MTZ, 1 µg), tetracycline (TET, 0.40 µg), SDS (0.25 µg) or Triton X-100 (0.53 µg) were placed on agar plates seeded with W50 or *pg1058* mutant cells. The zone of growth inhibition (mm) was measured after 24 h. Mean ± SEM; *N*=4; See S1 Experimental Procedures.
