# Supplementary material for: PG1058 Is a Novel Multidomain Protein Component of the Bacterial Type IX Secretion System
Source: PLoS One. 2016 Oct 6;11(10):e0164313. doi: 10.1371/journal.pone.0164313 (PMC5053529; doi:10.1371/journal.pone.0164313)
Supplement: S4 Fig — The combined Mascot score of confirmed non-CTD OM proteins (black bars) and IM proteins (white bars) within each fraction was compared to the total Mascot score for all proteins identified in that fraction as a percentage. N = 1. (DOCX) [file pone.0164313.s005.docx]

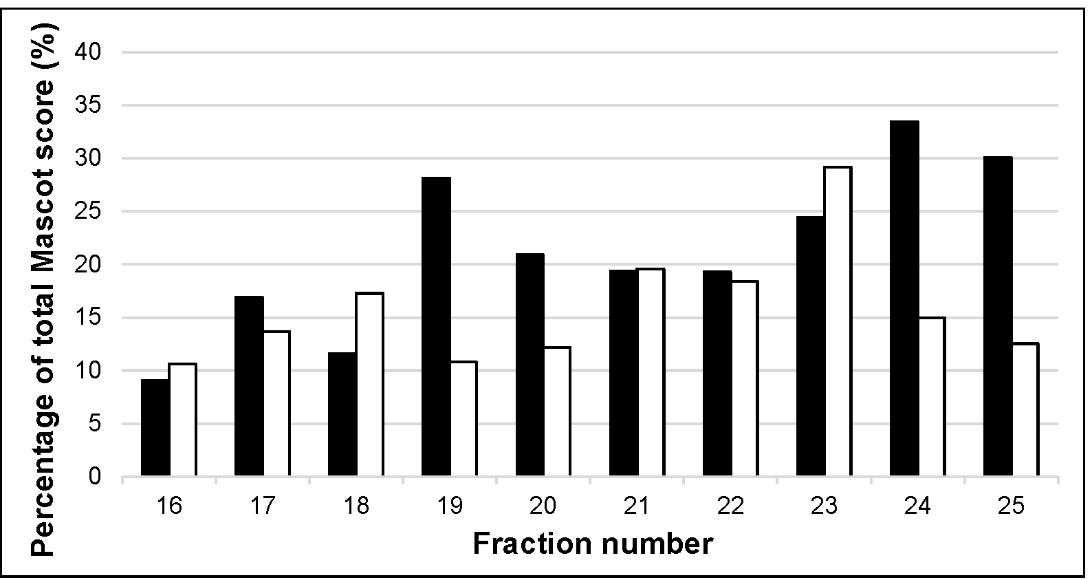


**S4 Fig. Proteomic analyses of isopycnic sucrose density gradient fractionation of the *P. gingivalis* total membrane.** The combined Mascot score of confirmed non-CTD OM proteins (black bars) and IM proteins (white bars) within each fraction was compared to the total Mascot score for all proteins identified in that fraction as a percentage. *N*=1.
