# Supplementary material for: PG1058 Is a Novel Multidomain Protein Component of the Bacterial Type IX Secretion System
Source: PLoS One. 2016 Oct 6;11(10):e0164313. doi: 10.1371/journal.pone.0164313 (PMC5053529; doi:10.1371/journal.pone.0164313)
Supplement: S1 Table — A, ATCC: American Type Culture Collection. B, Strain ECR669 has kgp from the Met codon to the stop codon substituted with cepA [89] that codes a cephalosporinase. This mutant was made by flanking cepA with >300 nt of DNA immediately 5’ and 3’ to kgp and using this to transform P. gingivalis W50. Replacement of kgp occurred after homologous recombination, with transformants selected on HBA supplemented with 5 μg/mL of ampicillin. (DOCX) [file pone.0164313.s006.docx]

**S1 Table. Bacterial Strains**

| **Strain/Plasmid** | **Relevant Genotype and Description** | **Source/Reference** |
| --- | --- | --- |
| *E. coli* α-Select |  | Bioline |
| *P. gingivalis* W50 | Wild-type | ATCC^a^ |
| *P. gingivalis* ECR370 | *pg1058^-^*; *pg1058*::*ermF* | This study |
| *P. gingivalis* ECR756 | *pg1058^+^*; *pg1058*::*ermF, mfa1*::[*pg1058^+^cepA*] | This study |
| *P. gingivalis* ECR669^b^ | *kgp^-^*; *kgp*::*cepA* | This study |

1. ATCC: American Type Culture Collection
2. Strain ECR669 has *kgp* from the Met codon to the stop codon substituted with *cepA* [[1](#_ENREF_1)] that codes a cephalosporinase. This mutant was made by flanking *cepA* with >300 nt of DNA immediately 5’ and 3’ to *kgp* and using this to transform *P. gingivalis* W50. Replacement of *kgp* occurred after homologous recombination, with transformants selected on HBA supplemented with 5 µg/mL of ampicillin.

**Reference**

1. Rogers MB, Bennett TK, Payne CM, Smith CJ. Insertional activation of *cepA* leads to high-level beta-lactamase expression in *Bacteroides fragilis* clinical isolates. Journal of Bacteriology. 1994;176(14):4376-84.
