# Supplementary material for: PG1058 Is a Novel Multidomain Protein Component of the Bacterial Type IX Secretion System
Source: PLoS One. 2016 Oct 6;11(10):e0164313. doi: 10.1371/journal.pone.0164313 (PMC5053529; doi:10.1371/journal.pone.0164313)
Supplement: S2 Table — (DOCX) [file pone.0164313.s007.docx]

**S2 Table. Oligonucleotide primers.**

| **Oligonucleotide** | **Sequence (5’→3’)** | **Comments** |
| --- | --- | --- |
| **Inactivation of *pg1058*** |  |  |
| PG1058_fwd1 | CCTGCAAGAGCGTGAAGTTG | *pg1058* forward primer*,* nt 68-87. |
| PG1058_rev1 | CGATCACACGGAACTCGGTAC | *pg1058* reverse primer, nt 2002-1982. |
| ***pg1058* Complementation** |  |  |
| cepAf | CGGATATAGG**GACGTC**AAAAGAG | GenBank accession BNRCEPA, nt 282-384; AatII site mutation (bold). |
| cepAr | GGCTACAGATACTG**GACGTC**TCAA | GenBank accession BNRCEPA , nt 1458-1435; AatII site mutation (bold). |
| PG0176ntigrNcoIFWD | CAGTCA**CCATGG**CTGAACACTCTATGTCGTGTCTTTG | Forward primer, region 5’ to *mfa1* (*pg0177*); NcoI site (bold). |
| PG0176LdrREsREV | CTGACT**CCGCGGCCGCCCGGGATCCATGCAT**AAGCCAAATG**TTTAAA**AGG**ATTAAT**ATTAAATTG | Reverse primer, region 5’ to *mfa1*; multiple cloning site: SacII, NotI, SmaI, BamHI, NsiI (bold). |
| PG0176GnSpeIFWD | CAGTCA**ACTAGT**ATGAAGTTAAACAAAATGTTTTTGGTCGGAGC | *pg1076* forward primer, nt 001-032; SpeI site (bold). |
| PG0176GnNdeIREV | CTGACT**CATATG**AGGCACCATGTAGATGCTCACTTTC | *pg1076* reverse primer, nt 246-222; NdeI site (bold). |
| PG1058compBamHIFor | caga**ggatcc**tacagcggacatcataaaatcc | *pg1058* ORF forward primer; BamHI site (bold) |
| PG1058compSacIIRev | caga**ccgcgg**gagcgattaacgcaactct | *pg1058* ORF reverse primer; SacII site (bold) |
| **RT-PCR** |  |  |
| PG1056For1 | cgcggagaatatggctgtttatc | *pg1056* forward primer, nt 294-316. |
| PG1057For2 | cacagccttcagggtgaag | *pg1057* forward primer, nt 22-40. |
| PG1058DomIFor1 | cagacccgggtttgctgcctgcaagagcgtg | *pg1058* forward primer, nt 61-81. |
| PG1058DomIRev1 | cagactcgagagaatccggatacccgtagcg | *pg1058* reverse primer, nt 294-274. |
| PG1056For2 | attacagccgaacgttatcacgac | *pg1056* forward primer, nt 4-27. |
| PG1056Rev2 | acagtcgcacgcaaagcag | *pg1056* reverse primer, nt 257-239. |
| PG1057Rev2 | ctccggtcttgggactaca | *pg1057* reverse primer, nt340-322. |
| PG1058DomIIIFor1 | cagacccgggaatatgggacaaccggtc | *pg1058* forward primer, nt 1144-1161. |
| PG1058DomIIIRev1 | cagactcgagcgatgcaaggaaaaagtccac | *pg1058* reverse primer, nt 1582-1561. |
