# Supplementary material for: PG1058 Is a Novel Multidomain Protein Component of the Bacterial Type IX Secretion System
Source: PLoS One. 2016 Oct 6;11(10):e0164313. doi: 10.1371/journal.pone.0164313 (PMC5053529; doi:10.1371/journal.pone.0164313)
Supplement: S3 Table — (DOCX) [file pone.0164313.s008.docx]

**S3 Table. *P. gingivalis* W50 protein localisation used for proteomic analyses.**

| **Locus Tag** | **Protein Description (Protein ID)** | **Localisation** | **Group** |
| --- | --- | --- | --- |
| PG0026 | hypothetical protein (PorU) | OM | Substrate |
| PG0182 | von Willebrand factor type A domain protein |  | Substrate |
| PG0183 | hypothetical protein |  | Substrate |
| PG0232 | zinc carboxypeptidase, putative | OM | Substrate |
| PG0350 | internalin-related protein | OM | Substrate |
| PG0411 | hemagglutinin, putative | OM | Substrate |
| PG0495 | hypothetical protein |  | Substrate |
| PG0506 | arginine-specific cysteine proteinase (RgpB) | OM | Substrate |
| PG0553 | extracellular protease, putative | OM | Substrate |
| PG0611 | hypothetical protein | PP | Substrate |
| PG0614 | hypothetical protein |  | Substrate |
| PG0616 | thioredoxin, putative | OM | Substrate |
| PG0626 | hypothetical protein | OM | Substrate |
| PG0654 | hypothetical protein | OM | Substrate |
| PG0769 | fibronectin type III domain protein |  | Substrate |
| PG0787 | hypothetical protein |  | Substrate |
| PG1030 | hypothetical protein | PP | Substrate |
| PG1326 | hemagglutinin, putative |  | Substrate |
| PG1374 | immunoreactive 47 kDa antigen PG97 | OM | Substrate |
| PG1424 | peptidylarginine deiminase (PAD) | OM | Substrate |
| PG1427 | thiol protease/hemagglutinin PrtT precursor, putative | OM | Substrate |
| PG1604 | immunoreactive 84 kDa antigen PG93 | PP | Substrate |
| PG1795 | hypothetical protein | OM | Substrate |
| PG1798 | immunoreactive 46 kDa antigen PG99 | OM | Substrate |
| PG1837 | hemagglutinin protein (HagA) | OM | Substrate |
| PG1844 | lysine-specific cysteine proteinase, authentic frameshift (Kgp) |  | Substrate |
| PG1969 | hypothetical protein | OM | Substrate |
| PG2024 | arginine-specific protease ArgI polyprotein (RgpA) | OM | Substrate |
| PG2100 | immunoreactive 63 kDa antigen PG102 | OM | Substrate |
| PG2102 | immunoreactive 61 kDa antigen PG91 | OM | Substrate |
| PG2172 | hypothetical protein |  | Substrate |
| PG2198 | immunoreactive 32 kDa antigen PG25 |  | Substrate |
| PG2216 | hypothetical protein | OM | Substrate |
| PG0027 | hypothetical protein (PorV/LptO) | OM | Component |
| PG0052 | sensor histidine kinase (PorY) |  | Component |
| PG0133 | hypothetical protein |  | Component |
| PG0162 | RNA polymerase sigma-70 factor, ECF subfamily |  | Component |
| PG0236 | hypothetical protein | PP | Component |
| PG0264 | glycosyl transferase, group 2 family protein |  | Component |
| PG0287 | hypothetical protein (PorP) |  | Component |
| PG0288 | lipoprotein, putative (PorK) | M | Component |
| PG0289 | hypothetical protein (PorL) | IM | Component |
| PG0290 | hypothetical protein (PorM) | IM | Component |
| PG0291 | hypothetical protein (PorN) |  | Component |
| PG0441 | hypothetical protein |  | Component |
| PG0534 | hypothetical protein |  | Component |
| PG0602 | hypothetical protein (PorQ) | OM | Component |
| PG0751 | porT protein (PorT) | OM | Component |
| PG0809 | hypothetical protein (Sov) |  | Component |
| PG0928 | response regulator (PorX) |  | Component |
| PG0945 | ABC transporter, permease protein, putative | M | Component |
| PG1058 | outer membrane protein | PP | Component |
| PG1571 | metallo-beta-lactamase superfamily protein |  | Component |
| PG1572 | membrane protein |  | Component |
| PG1573 | transcriptional regulator, Crp family |  | Component |
| PG1685 | hypothetical protein |  | Component |
| PG1786 | hypothetical protein | OM | Component |
| PG1850 | hypothetical protein | PP | Component |
| PG1947 | TPR domain protein (PorW) |  | Component |
| PG2071 | conserved domain protein |  | Component |
| PG2092 | hypothetical protein | PP | Component |
| PG0032 | beta-mannosidase, putative | PP | Periplasm |
| PG0196 | peptidase, M16 family | PP | Periplasm |
| PG0235 | carboxyl-terminal protease | PP | Periplasm |
| PG0320 | hypothetical protein | PP | Periplasm |
| PG0449 | TPR domain protein | PP | Periplasm |
| PG0491 | conserved hypothetical protein | PP | Periplasm |
| PG0503 | dipeptidyl aminopeptidase IV | PP | Periplasm |
| PG0724 | prolyl oligopeptidase family protein | PP | Periplasm |
| PG0890 | alkaline phosphatase, putative | PP | Periplasm |
| PG1004 | prolyl oligopeptidase family protein | PP | Periplasm |
| PG1226 | peptidyl-prolyl cis-trans isomerase, cyclophilin-type | PP | Periplasm |
| PG1313 | conserved domain protein | PP | Periplasm |
| PG1361 | dipeptidyl aminopeptidase IV, putative | PP | Periplasm |
| PG1385 | TPR domain protein | PP | Periplasm |
| PG1634 | hypothetical protein | PP | Periplasm |
| PG1726 | PDZ domain protein | PP | Periplasm |
| PG2083 | hypothetical protein | PP | Periplasm |
| PG2155 | hypothetical protein | PP | Periplasm |
| PG2175 | conserved hypothetical protein | PP | Periplasm |
| PG2227 | hypothetical protein | PP | Periplasm |
| PG0076 | N-acetylmuramoyl-L-alanine amidase, family 4 | OM | Total Membrane |
| PG0082 | hypothetical protein | OM | Total Membrane |
| PG0083 | hypothetical protein | OM | Total Membrane |
| PG0140 | hypothetical protein | OM | Total Membrane |
| PG0180 | hypothetical protein | OM | Total Membrane |
| PG0181 | immunoreactive 32 kDa antigen PG49 | OM | Total Membrane |
| PG0183 | hypothetical protein | OM | Total Membrane |
| PG0185 | ragA protein | OM | Total Membrane |
| PG0186 | lipoprotein RagB | OM | Total Membrane |
| PG0188 | hypothetical protein | OM | Total Membrane |
| PG0216 | hypothetical protein | OM | Total Membrane |
| PG0217 | hypothetical protein | OM | Total Membrane |
| PG0218 | hypothetical protein | OM | Total Membrane |
| PG0326 | hypothetical protein | OM | Total Membrane |
| PG0373 | hypothetical protein | OM | Total Membrane |
| PG0409 | hypothetical protein | OM | Total Membrane |
| PG0419 | hypothetical protein | OM | Total Membrane |
| PG0421 | hypothetical protein | OM | Total Membrane |
| PG0448 | hypothetical protein | OM | Total Membrane |
| PG0495 | hypothetical protein | OM | Total Membrane |
| PG0593 | HtrA protein | OM | Total Membrane |
| PG0668 | TonB-dependent receptor | OM | Total Membrane |
| PG0669 | heme-binding protein FetB | OM | Total Membrane |
| PG0694 | immunoreactive 42 kDa antigen PG33 | OM | Total Membrane |
| PG0695 | immunoreactive 43 kDa antigen PG32 | OM | Total Membrane |
| PG0706 | hypothetical protein | OM | Total Membrane |
| PG0707 | hypothetical protein | OM | Total Membrane |
| PG0726 | hypothetical protein | OM | Total Membrane |
| PG0937 | hypothetical protein | OM | Total Membrane |
| PG0955 | hypothetical protein | OM | Total Membrane |
| PG0987 | hypothetical protein | OM | Total Membrane |
| PG0TLR | TLR TonB-linked receptor BAMBU sequence | OM | Total Membrane |
| PG1093 | hypothetical protein | OM | Total Membrane |
| PG1185 | hypothetical protein | OM | Total Membrane |
| PG1215 | hypothetical protein | OM | Total Membrane |
| PG1341 | hypothetical protein | OM | Total Membrane |
| PG1382 | hypothetical protein | OM | Total Membrane |
| PG1414 | hypothetical protein | OM | Total Membrane |
| PG1551 | hmuY protein | OM | Total Membrane |
| PG1620 | carboxyl-terminal protease-related protein | OM | Total Membrane |
| PG1621 | hypothetical protein | OM | Total Membrane |
| PG1626 | hypothetical protein | OM | Total Membrane |
| PG1651 | TPR domain protein | OM | Total Membrane |
| PG1684 | hypothetical protein | OM | Total Membrane |
| PG1713 | lipoprotein, putative | OM | Total Membrane |
| PG1757 | hypothetical protein | OM | Total Membrane |
| PG1823 | hypothetical protein | OM | Total Membrane |
| PG1835 | hypothetical protein | OM | Total Membrane |
| PG1844 | lysine-specific cysteine proteinase, authentic frameshift | OM | Total Membrane |
| PG1881 | hypothetical protein | OM | Total Membrane |
| PG1889 | hypothetical protein | OM | Total Membrane |
| PG2008 | hypothetical protein | OM | Total Membrane |
| PG2041 | hypothetical protein | OM | Total Membrane |
| PG2054 | lipoprotein PG3 | OM | Total Membrane |
| PG2105 | hypothetical protein | OM | Total Membrane |
| PG2106 | hypothetical protein | OM | Total Membrane |
| PG2112 | hypothetical protein | OM | Total Membrane |
| PG2132 | fimbrilin | OM | Total Membrane |
| PG2149 | hypothetical protein | OM | Total Membrane |
| PG2164 | peptidyl-prolyl cis-trans isomerase, FKBP-type | OM | Total Membrane |
| PG2167 | immunoreactive 53 kDa antigen PG123 | OM | Total Membrane |
| PG2168 | hypothetical protein | OM | Total Membrane |
| PG2172 | hypothetical protein | OM | Total Membrane |
| PG2173 | outer membrane lipoprotein Omp28 | OM | Total Membrane |
| PG2197 | conserved hypothetical protein | OM | Total Membrane |
| PG0045 | heat shock protein HtpG | Membrane | Total Membrane |
| PG0093 | HlyD family secretion protein | Membrane | Total Membrane |
| PG0094 | outer membrane efflux protein, putative | Membrane | Total Membrane |
| PG0191 | outer membrane protein, putative | Membrane | Total Membrane |
| PG0226 | transglutaminase-related protein | Membrane | Total Membrane |
| PG0255 | translation initiation factor IF-2 | Membrane | Total Membrane |
| PG0276 | conserved hypothetical protein | Membrane | Total Membrane |
| PG0303 | iron-sulfur cluster binding protein | Membrane | Total Membrane |
| PG0423 | hypothetical protein | Membrane | Total Membrane |
| PG0437 | polysaccharide export protein, BexD/CtrA/VexA family | Membrane | Total Membrane |
| PG0620 | ATP-dependent protease La | Membrane | Total Membrane |
| PG0779 | hypothetical protein | Membrane | Total Membrane |
| PG0946 | ABC transporter, ATP-binding protein | Membrane | Total Membrane |
| PG1006 | hypothetical protein | Membrane | Total Membrane |
| PG1049 | conserved hypothetical protein | Membrane | Total Membrane |
| PG1080 | 3-hydroxyacyl-CoA dehydrogenase family protein | Membrane | Total Membrane |
| PG1139 | hypothetical protein | Membrane | Total Membrane |
| PG1140 | glycosyl transferase, group 2 family protein | Membrane | Total Membrane |
| PG1230 | hypothetical protein | Membrane | Total Membrane |
| PG1260 | anaerobic ribonucleoside-triphosphate reductase, putative | Membrane | Total Membrane |
| PG1367 | hypothetical protein | Membrane | Total Membrane |
| PG1379 | ABC transporter, periplasmic substrate-binding protein, putative | Membrane | Total Membrane |
| PG1396 | cell shape-determining protein MreB | Membrane | Total Membrane |
| PG1430 | TPR domain protein | Membrane | Total Membrane |
| PG1579 | ATPase, MoxR family | Membrane | Total Membrane |
| PG1591 | conserved hypothetical protein | Membrane | Total Membrane |
| PG1614 | fumarate reductase, iron-sulfur protein | Membrane | Total Membrane |
| PG1638 | thioredoxin family protein | Membrane | Total Membrane |
| PG1652 | hypothetical protein | Membrane | Total Membrane |
| PG1759 | adhesion protein, putative | Membrane | Total Membrane |
| PG1802 | hypothetical protein | Membrane | Total Membrane |
| PG1918 | preprotein translocase, SecY subunit | Membrane | Total Membrane |
| PG1978 | hypothetical protein | Membrane | Total Membrane |
| PG2001 | signal peptidase I | Membrane | Total Membrane |
| PG2029 | hypothetical protein | Membrane | Total Membrane |
| PG2144 | hypothetical protein | Membrane | Total Membrane |
| PG2177 | NADH:ubiquinone oxidoreductase, Na translocating, F subunit | Membrane | Total Membrane |
| PG2180 | NADH:ubiquinone oxidoreductase, Na translocating, C subunit | Membrane | Total Membrane |
| PG2182 | NADH:ubiquinone oxidoreductase, Na translocating, A subunit | Membrane | Total Membrane |
| PG2207 | conserved domain protein | Membrane | Total Membrane |
| PG0056 | hypothetical protein | IM | Total Membrane |
| PG0159 | endopeptidase PepO | IM | Total Membrane |
| PG0241 | hypothetical protein | IM | Total Membrane |
| PG0254 | N utilization substance protein A, putative | IM | Total Membrane |
| PG0304 | NADH dehydrogenase, 51 kDa subunit, putative | IM | Total Membrane |
| PG0306 | conserved hypothetical protein | IM | Total Membrane |
| PG0332 | transcription termination factor Rho | IM | Total Membrane |
| PG0360 | lemA protein | IM | Total Membrane |
| PG0377 | ribosomal protein S2 | IM | Total Membrane |
| PG0401 | KH/HDIG domain protein | IM | Total Membrane |
| PG0452 | hypothetical protein | IM | Total Membrane |
| PG0514 | preprotein translocase, SecA subunit | IM | Total Membrane |
| PG0535 | conserved hypothetical protein | IM | Total Membrane |
| PG0639 | signal peptide peptidase SppA, 67K type | IM | Total Membrane |
| PG0685 | ABC transporter, ATP-binding protein | IM | Total Membrane |
| PG0708 | peptidyl-prolyl cis-trans isomerase, FKBP-type, putative | IM | Total Membrane |
| PG0758 | peptidyl-dipeptidase Dcp | IM | Total Membrane |
| PG0780 | hypothetical protein | IM | Total Membrane |
| PG0782 | MotA/TolQ/ExbB proton channel family protein | IM | Total Membrane |
| PG1010 | ABC transporter, ATP-binding protein | IM | Total Membrane |
| PG1084 | thioredoxin family protein | IM | Total Membrane |
| PG1115 | signal recognition particle protein | IM | Total Membrane |
| PG1129 | ribonucleotide reductase | IM | Total Membrane |
| PG1302 | hypothetical protein | IM | Total Membrane |
| PG1330 | large conductance mechanosensitive channel protein | IM | Total Membrane |
| PG1334 | band 7/Mec-2 family protein | IM | Total Membrane |
| PG1608 | methylmalonyl-CoA decarboxylase, beta subunit | IM | Total Membrane |
| PG1611 | hypothetical protein | IM | Total Membrane |
| PG1615 | fumarate reductase, flavoprotein subunit | IM | Total Membrane |
| PG1704 | thiol:disulfide interchange protein dsbD, putative | IM | Total Membrane |
| PG1762 | protein-export membrane protein SecD/protein-export membrane protein SecF | IM | Total Membrane |
| PG1803 | v-type ATPase, subunit A | IM | Total Membrane |
| PG1804 | v-type ATPase, subunit B | IM | Total Membrane |
| PG2050 | hypothetical protein | IM | Total Membrane |
| PG2082 | POT family protein | IM | Total Membrane |
| PG2174 | hypothetical protein | IM | Total Membrane |
| PG0034 | thioredoxin | Cytoplasm | Cytoplasm |
| PG0042 | serine hydroxymethyltransferase | Cytoplasm | Cytoplasm |
| PG0090 | Dps family protein | Cytoplasm | Cytoplasm |
| PG0130 | phosphoglycerate mutase | Cytoplasm | Cytoplasm |
| PG0153 | aspartyl-tRNA synthetase | Cytoplasm | Cytoplasm |
| PG0195 | rubrerythrin | Cytoplasm | Cytoplasm |
| PG0296 | phosphoribosylformylglycinamidine synthase | Cytoplasm | Cytoplasm |
| PG0316 | seryl-tRNA synthetase | Cytoplasm | Cytoplasm |
| PG0324 | histidine ammonia-lyase | Cytoplasm | Cytoplasm |
| PG0329 | formiminotransferase-cyclodeaminase-related protein | Cytoplasm | Cytoplasm |
| PG0330 | DNA-binding protein, histone-like family | Cytoplasm | Cytoplasm |
| PG0343 | methionine gamma-lyase, putative | Cytoplasm | Cytoplasm |
| PG0378 | translation elongation factor Ts | Cytoplasm | Cytoplasm |
| PG0386 | site-specific recombinase, phage integrase family / ribosomal subunit interface protein | Cytoplasm | Cytoplasm |
| PG0418 | ATP-dependent Clp protease, proteolytic subunit | Cytoplasm | Cytoplasm |
| PG0481 | 8-amino-7-oxononanoate synthase | Cytoplasm | Cytoplasm |
| PG0482 | hypothetical protein | Cytoplasm | Cytoplasm |
| PG0520 | chaperonin, 60 kDa | Cytoplasm | Cytoplasm |
| PG0521 | chaperonin, 10 kDa | Cytoplasm | Cytoplasm |
| PG0523 | inosine-5'-monophosphate dehydrogenase | Cytoplasm | Cytoplasm |
| PG0537 | aminoacyl-histidine dipeptidase | Cytoplasm | Cytoplasm |
| PG0548 | pyruvate ferredoxin/flavodoxin oxidoreductase family protein | Cytoplasm | Cytoplasm |
| PG0558 | purine nucleoside phosphorylase, family 2 | Cytoplasm | Cytoplasm |
| PG0561 | peptidase, M20/M25/M40 family | Cytoplasm | Cytoplasm |
| PG0571 | aspartate-semialdehyde dehydrogenase | Cytoplasm | Cytoplasm |
| PG0589 | GMP synthase | Cytoplasm | Cytoplasm |
| PG0595 | ribosomal protein S6 | Cytoplasm | Cytoplasm |
| PG0623 | triosephosphate isomerase | Cytoplasm | Cytoplasm |
| PG0634 | ThiJ/PfpI family protein | Cytoplasm | Cytoplasm |
| PG0687 | succinate-semialdehyde dehydrogenase | Cytoplasm | Cytoplasm |
| PG0689 | NAD-dependent 4-hydroxybutyrate dehydrogenase | Cytoplasm | Cytoplasm |
| PG0690 | 4-hydroxybutyrate CoA-transferase | Cytoplasm | Cytoplasm |
| PG0692 | 4-hydroxybutyryl-CoA dehydratase | Cytoplasm | Cytoplasm |
| PG0733 | riboflavin synthase, alpha subunit | Cytoplasm | Cytoplasm |
| PG0762 | trigger factor, putative | Cytoplasm | Cytoplasm |
| PG0775 | acyl-CoA dehydrogenase family protein | Cytoplasm | Cytoplasm |
| PG0776 | electron transfer flavoprotein, alpha subunit | Cytoplasm | Cytoplasm |
| PG0791 | adenylate kinase | Cytoplasm | Cytoplasm |
| PG0793 | fructose-1,6-bisphosphatase | Cytoplasm | Cytoplasm |
| PG0802 | alpha keto acid dehydrogenase complex, E3 component, lipoamide dehydrogenase | Cytoplasm | Cytoplasm |
| PG0804 | flavodoxin | Cytoplasm | Cytoplasm |
| PG0933 | translation elongation factor G, putative | Cytoplasm | Cytoplasm |
| PG0976 | phosphoribosylaminoimidazole-succinocarboxamide synthase, putative | Cytoplasm | Cytoplasm |
| PG1066 | butyrate-acetoacetate CoA-transferase, subunit A | Cytoplasm | Cytoplasm |
| PG1069 | alcohol dehydrogenase, zinc-containing, putative | Cytoplasm | Cytoplasm |
| PG1073 | D-lysine 5,6-aminomutase, alpha subunit | Cytoplasm | Cytoplasm |
| PG1076 | acyl-CoA dehydrogenase, short-chain specific | Cytoplasm | Cytoplasm |
| PG1078 | electron transfer flavoprotein, alpha subunit | Cytoplasm | Cytoplasm |
| PG1081 | acetate kinase | Cytoplasm | Cytoplasm |
| PG1082 | phosphotransacetylase | Cytoplasm | Cytoplasm |
| PG1085 | hypothetical protein | Cytoplasm | Cytoplasm |
| PG1116 | methylenetetrahydrofolate dehydrogenase/methenyltetrahydrofolate cyclohydrolase | Cytoplasm | Cytoplasm |
| PG1123 | adenylosuccinate lyase | Cytoplasm | Cytoplasm |
| PG1134 | thioredoxin reductase | Cytoplasm | Cytoplasm |
| PG1189 | hypothetical protein | Cytoplasm | Cytoplasm |
| PG1208 | dnaK protein | Cytoplasm | Cytoplasm |
| PG1210 | peptidase, M24 family protein | Cytoplasm | Cytoplasm |
| PG1232 | glutamate dehydrogenase, NAD-specific | Cytoplasm | Cytoplasm |
| PG1235 | epimerase/reductase, putative | Cytoplasm | Cytoplasm |
| PG1269 | delta-1-pyrroline-5-carboxylate dehydrogenase | Cytoplasm | Cytoplasm |
| PG1271 | acetylornithine aminotransferase, putative | Cytoplasm | Cytoplasm |
| PG1280 | conserved hypothetical protein | Cytoplasm | Cytoplasm |
| PG1286 | ferritin | Cytoplasm | Cytoplasm |
| PG1290 | branched-chain amino acid aminotransferase | Cytoplasm | Cytoplasm |
| PG1305 | glycine cleavage system P protein | Cytoplasm | Cytoplasm |
| PG1321 | formate--tetrahydrofolate ligase | Cytoplasm | Cytoplasm |
| PG1327 | aminotransferase, putative | Cytoplasm | Cytoplasm |
| PG1353 | orotate phosphoribosyltransferase | Cytoplasm | Cytoplasm |
| PG1397 | phosphoribosylaminoimidazolecarboxamide formyltransferase/IMP cyclohydrolase | Cytoplasm | Cytoplasm |
| PG1401 | beta-eliminating lyase | Cytoplasm | Cytoplasm |
| PG1417 | fumarate hydratase class I, anaerobic | Cytoplasm | Cytoplasm |
| PG1540 | S-adenosylmethionine:tRNA ribosyltransferase-isomerase | Cytoplasm | Cytoplasm |
| PG1545 | superoxide dismutase, Fe-Mn | Cytoplasm | Cytoplasm |
| PG1559 | glycine cleavage system T protein | Cytoplasm | Cytoplasm |
| PG1656 | methylmalonyl-CoA mutase, small subunit | Cytoplasm | Cytoplasm |
| PG1657 | methylmalonyl-CoA mutase, large subunit | Cytoplasm | Cytoplasm |
| PG1676 | phosphoenolpyruvate carboxykinase (ATP) | Cytoplasm | Cytoplasm |
| PG1677 | phosphoglycerate kinase | Cytoplasm | Cytoplasm |
| PG1721 | ribonuclease R | Cytoplasm | Cytoplasm |
| PG1741 | aspartate ammonia-lyase | Cytoplasm | Cytoplasm |
| PG1764 | 3-oxoacyl-(acyl-carrier-protein) synthase II | Cytoplasm | Cytoplasm |
| PG1775 | grpE protein | Cytoplasm | Cytoplasm |
| PG1816 | NAD(P)H dehydrogenase, quinone family, putative | Cytoplasm | Cytoplasm |
| PG1824 | enolase | Cytoplasm | Cytoplasm |
| PG1847 | endoribonuclease L-PSP, putative | Cytoplasm | Cytoplasm |
| PG1853 | DNA polymerase III, beta subunit | Cytoplasm | Cytoplasm |
| PG1872 | urocanate hydratase | Cytoplasm | Cytoplasm |
| PG1914 | ribosomal protein S13 | Cytoplasm | Cytoplasm |
| PG1924 | ribosomal protein S8 | Cytoplasm | Cytoplasm |
| PG1951 | glutaminyl-tRNA synthetase | Cytoplasm | Cytoplasm |
| PG1975 | hemagglutinin protein HagC | Cytoplasm | Cytoplasm |
| PG2097 | ribose-phosphate pyrophosphokinase | Cytoplasm | Cytoplasm |
| PG2124 | glyceraldehyde 3-phosphate dehydrogenase | Cytoplasm | Cytoplasm |
